# Exploring the resistome, virulome, and mobilome of multidrug-resistant *Klebsiella pneumoniae* isolates: deciphering the molecular basis of carbapenem resistance

**DOI:** 10.1186/s12864-024-10139-y

**Published:** 2024-04-25

**Authors:** Sidra Rahmat Ullah, Sidra Irum, Iqra Mahnoor, Humaira Ismatullah, Mariam Mumtaz, Saadia Andleeb, Abdur Rahman, Muhsin Jamal

**Affiliations:** 1 Atta-ur-Rahman School of Applied Biosciences (ASAB), National University of Sciences and Technology, Islamabad, Pakistan; 2 Research Centre for Modelling & Simulation (RCMS), National University of Sciences and Technology, Islamabad, Pakistan; 3 Department of Microbiology, Abdul Wali Khan University, Mardan, Pakistan

**Keywords:** *Klebsiella pneumoniae*, Whole genome sequencing, Comparative genomics, Carbapenem resistance, Resistome, Mobilome, Virulome, Pakistan, ARGs

## Abstract

**Background:**

*Klebsiella pneumoniae*, a notorious pathogen for causing nosocomial infections has become a major cause of neonatal septicemia, leading to high morbidity and mortality worldwide. This opportunistic bacterium has become highly resistant to antibiotics due to the widespread acquisition of genes encoding a variety of enzymes such as extended-spectrum beta-lactamases (ESBLs) and carbapenemases. We collected *Klebsiella pneumoniae* isolates from a local tertiary care hospital from February 2019–February 2021. To gain molecular insight into the resistome, virulome, and genetic environment of significant genes of multidrug-resistant *K. pneumoniae* isolates, we performed the short-read whole-genome sequencing of 10 *K. pneumoniae* isolates recovered from adult patients, neonates, and hospital tap water samples.

**Results:**

The draft genomes of the isolates varied in size, ranging from 5.48 to 5.96 Mbp suggesting the genome plasticity of this pathogen. Various genes conferring resistance to different classes of antibiotics e.g., aminoglycosides, quinolones, sulfonamides, tetracycline, and trimethoprim were identified in all sequenced isolates. The highest resistance was observed towards carbapenems, which has been putatively linked to the presence of both class B and class D carbapenemases, *bla*_*NDM,*_ and *bla*_*OXA*_, respectively. Moreover, the biocide resistance gene *qacEdelta1* was found in 6/10 of the sequenced strains. The sequenced isolates exhibited a broad range of sequence types and capsular types. The significant antibiotic resistance genes (ARGs) were bracketed by a variety of mobile genetic elements (MGEs). Various spontaneous mutations in genes other than the acquired antibiotic-resistance genes were observed, which play an indirect role in making these bugs resistant to antibiotics*.* Loss or deficiency of outer membrane porins, combined with ESBL production, played a significant role in carbapenem resistance in our sequenced isolates. Phylogenetic analysis revealed that the study isolates exhibited evolutionary relationships with strains from China, India, and the USA suggesting a shared evolutionary history and potential dissemination of similar genes amongst the isolates of different origins.

**Conclusions:**

This study provides valuable insight into the presence of multiple mechanisms of carbapenem resistance in *K. pneumoniae* strains including the acquisition of multiple antibiotic-resistance genes through mobile genetic elements. Identification of rich mobilome yielded insightful information regarding the crucial role of insertion sequences, transposons, and integrons in shaping the genome of bacteria for the transmission of various resistance-associated genes. Multi-drug resistant isolates that had the fewest resistance genes exhibited a significant number of mutations. *K. pneumoniae* isolate from water source displayed comparable antibiotic resistance determinants to clinical isolates and the highest number of virulence-associated genes suggesting the possible interplay of ARGs amongst bacteria from different sources.

**Supplementary Information:**

The online version contains supplementary material available at 10.1186/s12864-024-10139-y.

## Introduction

*Klebsiella pneumoniae* (*K. pneumoniae*) is an important member of the *Enterobacteriaceae* family and a natural inhabitant of the gastrointestinal tract (GIT) microflora of humans and animals. It has evolved as an “antimicrobial resistant opportunist” accountable for one-third of all Gram-negative infections [1–3]. This bacteria lies in the list of ESKAPE organism amongst “critical priority pathogens” by the World Health Organization (WHO) [4]. *K. pneumoniae* has risen as a significant pathogen in Neonatal Intensive Care Units (NICUs) in developing countries having a mortality rate of 18–68% [5, 6]. The NICU-admitted and premature newborns face a higher susceptibility to risk due to their underdeveloped immune system, unsettled microflora, and slightly enhanced permeability of the GIT mucosa [7]. Over the past decade, a significant increase has been observed in sepsis and bacteremia in the neonatal population due to multi-drug resistant bacteria, globally [8]. With the ongoing pandemic, the situation has become increasingly perilous. Recent reports indicate that a considerable proportion of COVID-19 patients, with a mortality rate of 56.7%, are experiencing secondary bacterial infections. Among these infections, the predominant pathogen isolated from all affected individuals is MDR *K. pneumoniae* [9]. Despite the appropriate use of antimicrobial therapy, fatality rates have risen to 50% making *K. pneumoniae* the leading cause of mortality and morbidity by causing nosocomial infections [10]. The spread of antibiotic resistance genes (ARGs) in bacterial populations, is facilitated by horizontal gene transfer (HGT) aided by mobile genetic elements (MGEs). These MGEs encompass plasmids, transposons, and integrons. These elements serve as a carrier for ARGs and enable their transfer amongst different Gram-negative and Gram-positive bacterial species [11].

The rise of *K. pneumoniae* strains producing extended-spectrum β-lactamases (ESBLs), *Klebsiella pneumoniae* carbapenemases (KPCs), and Metallo β-lactamases (MBL) has become a pressing concern. The two primary non-specific porins produced by *K. pneumoniae* are OmpK35 and OmpK36, which facilitate the passive diffusion of small hydrophilic molecules and clinically important antibiotics like β-lactams and fluoroquinolones. Mutations in these outer membrane porins are linked with carbapenemase enzymes to enhance carbapenem resistance in this pathogen. Factors such as point mutations or insertional interruptions in the coding sequences or the promoter region can modify the expression of porins in clinical isolates of *K. pneumoniae*. Strains lacking both OmpK35 and OmpK36 have demonstrated elevated levels of antibiotic resistance [12, 13]. These resistant strains pose a substantial challenge in the clinical management of infections, as they are increasingly becoming untreatable [14]. According to the CDC Antimicrobial Resistance (AMR) action plan, tracking antimicrobial-resistant organisms is one of the four core actions proposed to limit the emergence and spread of multi-drug-resistant (MDR) pathogens [15]. To combat the frequent threat of outbreaks due to multi-drug resistant bacteria in immunocompromised patients in hospital settings, whole-genome sequencing (WGS) has become an important tool. With *K. pneumoniae* being established as an urgent and major health problem globally the WGS has become the most reliable tool for investigation and keeping track of bacterial pathogens [16]. Whole genome analysis of a pathogen can provide insights into its related lineages, pathogenicity, virulence, and antimicrobial resistance mechanisms that can revolutionize outbreak analysis and management [17].

We aim to perform a comprehensive genomics analysis to check the diversity of virulence-associated genes, antibiotic resistance genes (particularly involved in conferring resistance to last-resort antibiotics), and mobile genetic elements responsible for the dissemination of significant genes involved in drug resistance amongst *K. pneumoniae* strains recovered from different sources.

## Results

### AST Profile & Biofilm Assay

Antimicrobial susceptibility testing was carried out following Clinical and Laboratory Standards Institute (CLSI) 2018 guidelines. Based on the acquired antibiotic resistance profiles, 19% (41/214) strains were classified as susceptible, other 35% (75/214) strains as multi-drug-resistant (MDR), and the remaining 46% (99/214) strains as extensively drug resistant (XDR). Most of the isolates exhibited resistance to beta-lactam antibiotics. Resistance to fluoroquinolones and aminoglycosides was also observed in the majority of *K. pneumoniae* strains. The isolates were found susceptible to fosfomycin and did not exhibit any resistance towards polymyxin.

Based on the AST profiles generated using multi-resistance classification guidelines [18], four XDR and six MDR *K. pneumoniae* strains isolated from adults, neonates, and hospital tap water were subjected to whole genome sequencing. The biofilm detection assay revealed all XDR and one MDR strain (kp10) as excellent biofilm-former regardless of their source (Table 1). The AST profiles of our sequenced isolates are illustrated in Table 2.

**Table 1:** Source, host age, and biofilm formation ability of selected *Klebsiella pneumoniae* isolates

Strains	Year of Collection	Source	Host Age	Biofilm Former
kp5	2018	Blood	10 Days	Excellent
kp6	2018	Urine	27 years	Excellent
kp10	2018	Hospital Tap Water	NA	Excellent
kp47	2020	Blood	New Born	Excellent
kp54	2020	Blood	New Born	Excellent
kp58	2021	Blood	2 Days	Excellent
kp106	2018	Pus	37 years	Excellent
kp126	2019	Blood	5 Days	Moderate
kp127	2019	Blood	2 Days	Moderate
kp128	2019	Blood	3 Days	Moderate

**Table 2:** AST profile of the selected *Klebsiella pneumoniae* isolates

Strain	β lactams							Aminoglycosides			Fluoroquinolones				Combinations		Fosfomycin	Polymyxin	
	Penicillin	Cephalosporins				Carbapenems													
	AMP	FEP	CAZ	CTX	CRO	MEM	IMP	AK	CN	TOB	CIP	LEV	NOR	OFX	TZP	SXT	FOS	CT	
kp5	R	R	R	R	R	R	R	R	S	R	R	S	R	R	R	R	R	S	**XDR**
kp6	R	R	R	R	R	R	R	R	R	R	R	R	R	R	R	R	S	S	**XDR**
kp10	R	R	R	R	R	R	S	S	I	I	R	R	R	R	I	R	S	S	**MDR**
kp47	R	R	R	R	R	R	R	R	R	R	R	R	R	I	R	R	S	S	**MDR**
kp54	R	R	R	R	R	R	R	R	R	R	R	R	R	R	R	R	S	S	**XDR**
kp58	R	R	R	R	R	R	R	S	S	R	R	R	R	R	R	R	S	S	**MDR**
kp106	R	R	R	R	R	R	R	R	R	R	R	R	R	R	R	R	S	S	**XDR**
kp126	R	R	R	R	R	R	R	S	S	S	I	I	S	R	R	R	S	S	**MDR**
kp127	R	R	R	R	R	R	R	S	S	S	S	I	I	R	R	R	S	S	**MDR**
kp128	R	R	R	R	R	R	R	R	R	S	I	I	R	R	R	R	S	S	**MDR**

*AMP* Ampicillin, *FEP* Cefepime, *CAZ* Ceftazidime, *CTX* Cefotaxime, *CRO* Ceftriaxone, *MEM* Meropenem, *IMP* Imipenem, *AK* Amikacin, *CN* Gentamicin, *TOB* Tobramycin, *CIP* Ciprofloxacin, *LEV* Levofloxacin, *NOR* Norfloxacin, *OFX* Ofloxacin, *TZP* Ceftolozane/Tazobactam, *SXT* Trimethoprim/Sulfamethoxazole, *FOS* Fosfomycin, *CT* Colistin. Sensitive (Probably susceptible to ordinary dosage therapy), *I*: Intermediate (Likely to respond to high dosage therapy), *R*: Resistant (Unlikely to respond to high dosage therapy

### Genome characterizations

The draft genome sizes of the isolates ranged from 5.48 to 5.96 Mbp. The specific values for L50, N50, and contig numbers are shown in Supplementary Table 2. The water isolate kp10 exhibited the largest genome size, measuring 5.96 Mbp, in comparison to the genome sizes of other clinical isolates. The Whole Genome Shotgun project of the individual isolate has been deposited at DDBJ/ENA/GenBank under the accession numbers and the version described in this paper are described in Supplementary Table 1.

A comparative genomic analysis using BRIG was conducted to visually compare our sequenced isolates with the reference *K. pneumoniae* genome HS11286 [19] (Supplementary Fig. 1). The visual representation allowed a comprehensive comparison between the sequenced isolates and the reference genome, aiding in the identification of similarities, differences, and potential genomic variations.

### MLST and capsular types

The in silico MLST analysis revealed that the isolates encompassed seven distinct sequence types. Subsequent eBURST analysis assigned the sequenced strains to their respective clonal groups (CG). The study identified globally significant sequence types and recognized problematic clonal groups, such as water isolate kp10 falling within ST15/CG15 and clinical isolate kp47 in ST70/CG70 [1, 20–22]. *K. pneumoniae* typing is based on sequence variations of capsular polysaccharides known as K antigens and lipopolysaccharides such as O antigens. (2, 27). Within our study isolates, we noted the occurrence of four distinct O capsule types and identified eight distinct K capsule types, as presented in Table 3.

**Table 3:** MLST and Capsular typing of the selected *Klebsiella pneumoniae* isolates

Strains	MLST	K Antigen	O Antigen	Clonal Group (CG)
kp5	ST54	KL14	O3b	CG54
kp6	ST54	KL14	O3b	CG54
kp10	ST15	KL24	O1v1	CG15
kp47	ST70	KL22	O1/O2v2	CG70
kp54	ST29	KL149	O3b	CG29
kp58	ST2703	KL149	O3b	CG586
kp106	ST231	KL51	O1v2	CG231
kp126	ST22	KL9	O2v2	CG22
kp127	ST22	KL9	O2v2	CG22
kp128	ST22	KL9	O2v2	CG22

### Resistome of *K. pneumoniae* isolates

**Fig. 1:**
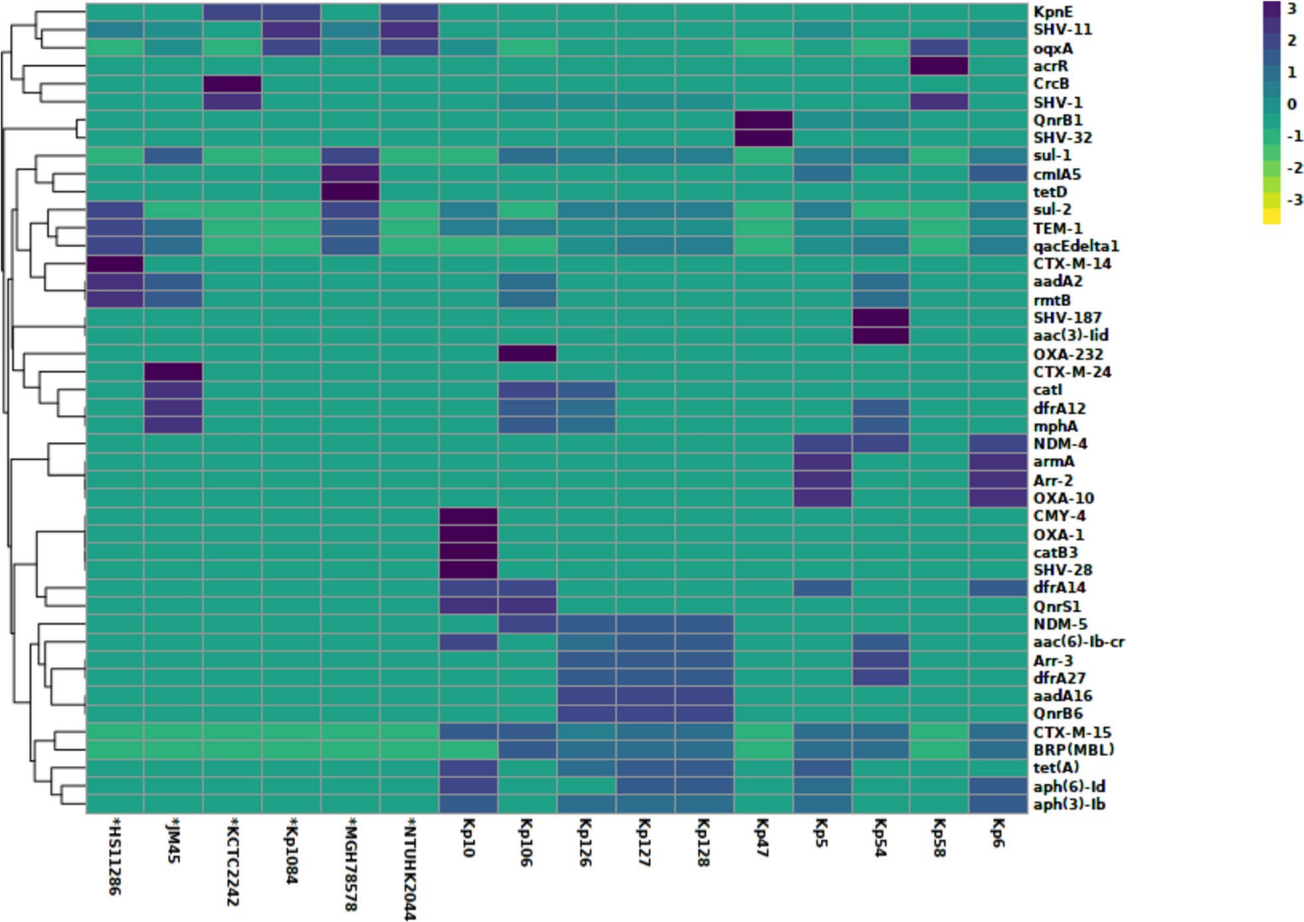
The heatmap displays the relative copy numbers of the ARGs. AMR determinants in *K. pneumoniae* genomes were clustered based on Euclidean distance and hierarchical ward algorithm. Sequenced isolates along with the reference genomes (Kp1084, HS11286, KCTC2242, MGH78578, NTUH2044 and JM45) are arranged on the X axis, while the resistance genes are plotted on the Y- axis. Resistance genes with a darker blue color have a relatively low copy number, while resistance genes with a lighter blue-yellow color have a relatively high copy number in comparison to others

Numerous antibiotic-resistance genes were observed in the sequenced *K. pneumoniae* isolates (Fig. 1). Further mining of whole-genome sequences revealed diverse β-lactamase encoding genes in the isolates. The majority of the isolates harbored more than two β-lactamase encoding genes in their genomes i.e. *bla*_*CTX-M-15*_*, bla*_*TEM,*_ *and bla*_*SHV*_. The highest resistance was observed towards carbapenems, which has been putatively linked to the presence of both class B and class D carbapenemases, *bla*_*NDM,*_ and *bla*_*OXA*_, respectively. The water isolate kp10 was the sole isolate containing the CMY-4 beta-lactamase, in addition to other resistance genes. The second most frequent group of resistance genes belonged to the *arr-2* and *arr-3* gene families against aminoglycosides. The plasmid-mediated quinolone resistance (PMQR) genes *oqx and qnr* were also detected amongst which *qnrB1, qnrB6,* and *qnrS1* were more prominent. The resistance observed towards trimethoprim-sulfamethoxazole antibiotics appears to be potentially linked to the presence of *sul1, sul2, and dfrA* genes. In addition, *tetA* and *tetD* genes were observed in the genomes of three of our isolates which have been reported to confer resistance to tetracycline. In all isolates, the biocide-resistance gene *qacEΔ1* was commonly observed. Interestingly, kp47 and kp58 exhibited phenotypic resistance to various antibiotics despite possessing the fewest number of resistance genes. On the contrary, no genetic determinant was found against colistin in any of the isolates. The correlation of genotypic and phenotypic data of major antibiotics is provided in Table 4.

**Table 4:** Correlation between genotypic and phenotypic resistance profiles of sequenced *K. pneumoniae* isolates

Strain	β Lactams		Carbapenem		Aminoglycosides		Fluoroquinolones		Miscellaneous	
	Genotype	Phenotype	Genotype	Phenotype	Genotype	Phenotype	Genotype	Phenotype	Genotype	Phenotype
**kp5**	SHV-11, CTX-M-15, TEM-1,	Resistant	NDM-4, OXA-10	Resistant	armA, aph(6)-Id, aph(3″)-Ib	Resistant to all except CN	QnrB1, oqxA	Resistant to all except LEV	sul1, sul2, dfrA12, tetA	Resistant
**kp6**	SHV-11, TEM-1,	Resistant	NDM-4, OXA-10	Resistant	armA	Resistant	oqxA	Resistant	sul1, sul2,	Resistant
**kp10**	SHV-28,, TEM-1, CTX-M-15,	Resistant	OXA-1, CMY-4	Resistant to MEM Susceptible to IMP	aph(3″)-Ib, aph(6)-Id, aac(6′)-Ib-cr	Intermediate Susceptible	QnrS1, aac(6′)-Ib-cr, oqxA	Resistant	sul-2, dfrA14, tetA	Resistant
**kp47**	SHV-32	Resistant	Nil	Resistant	Nil	Resistant	QnrB1	Resistant	KpnF	Resistant
**kp54**	SHV-187, CTX-M-15, TEM 1,	Resistant	NDM 1	Resistant	aac(3)-Iid, aadA2, rmtB	Resistant	aac(6′)-Ib-cr	Resistant	sul 1, drfA12, dfrA27	Resistant
**kp58**	SHV-1	Resistant	Nil	Resistant	Nil	Susceptible to AK, CN Resistant to TOB	oqxA	Resistant	Nil	Resistant
**kp106**	SHV-1, TEM-1, CTX-M-15	Resistant	OXA-232, NDM-5,	Resistant	rmtB, aadA2	Resistant	QnrS1	Resistant	sul1, dfrA12	Resistant
**kp126**	SHV-1, CTX-M-15,TEM-1,	Resistant	NDM-5	Resistant	Nil	Susceptible to AK, CN Intermediate to TOB	QnrB6	Intermediate	dfrA12, sul 1, sul 2	Resistant
**kp127**	SHV-1, CTX-M-15, TEM-1,	Resistant	NDM-5	Resistant	aac(6′)-Ib-cr, aph(3″)-Ib, aph(6)-Id	Susceptible	QnrB6, aac(6′)-Ib-cr	Intermediate	sul-1, sul-2	Resistant
**kp128**	SHV-1, CTX-M-15,,TEM-1,	Resistant	NDM-5	Resistant	aac(6′)-Ib-cr, aph(3″)-Ib, aph(6)-Id	Resistant	QnrB6, aac(6′)-Ib-cr	Resistant	sul-1, sul-2	Resistant

### Virulome of *K. pneumoniae* isolates

**Fig. 2:**
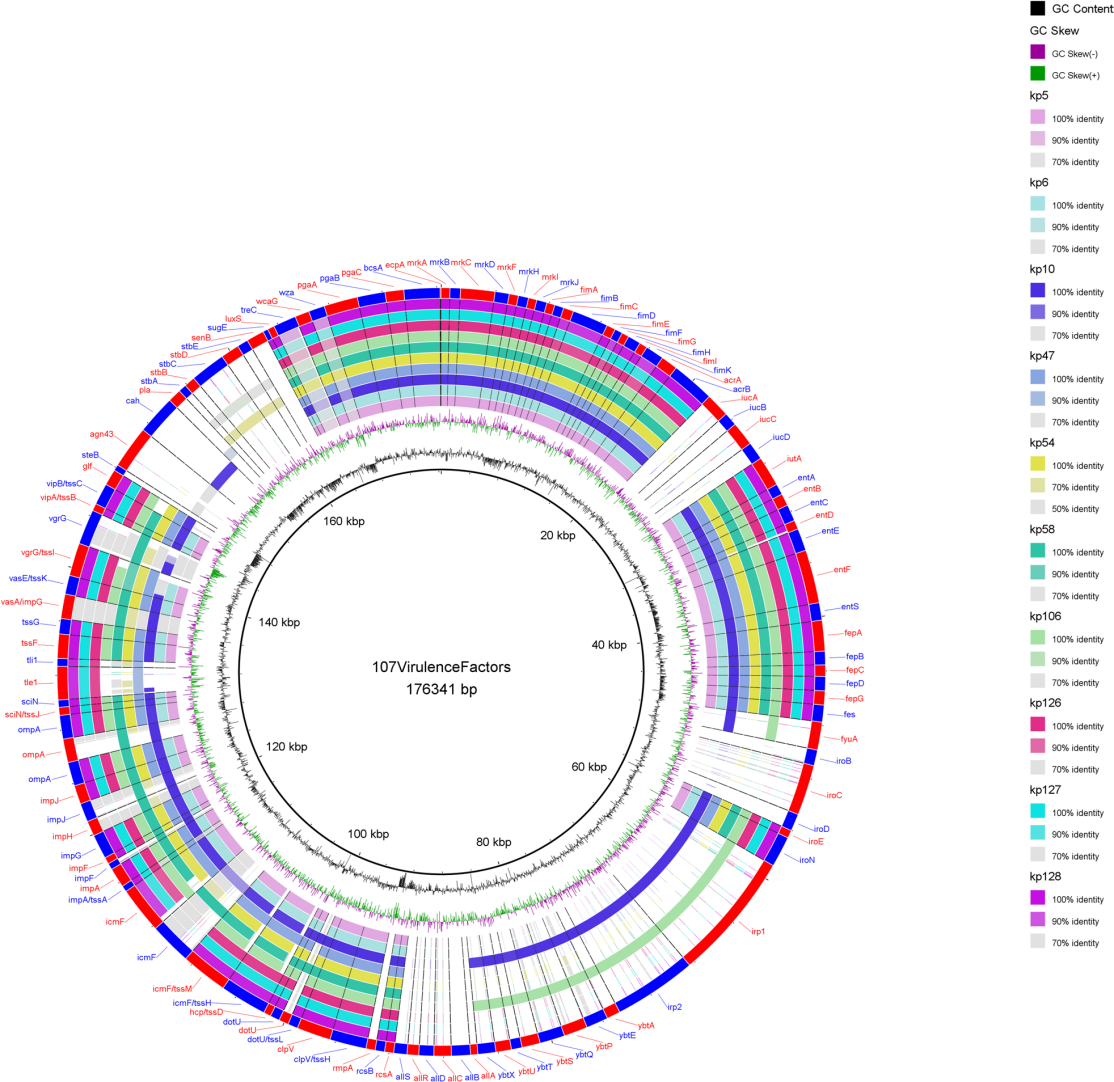
A circular diagram illustrating the whole genomes (chromosomes and plasmids) of this study. The draft genomes were aligned with 107 virulence genes from reference strains curated from VFDB, to assess their presence and distribution (Kp1084, HS11286, KCTC2242, MGH78578, NTUH2044 and JM45). Each genome is visually represented by a ring in the circular diagram, where different colors were used to indicate the percentage identity of the virulence gene in the studied genomes compared to the reference sequence. The color coding provides a visual representation of the degree of similarity or variation in the virulence genes across the genomes. The isolates kp10 and kp106 harbored the highest number of virulence factors. The image was generated using BRIG (http://brig.sourceforge.net)

Several genes associated with virulence factors, including adherence, capsule formation, efflux pumps, siderophores, type VI secretion system (T6SS-III), serum resistance, and immune evasion, were identified in all isolates of our study. Nonetheless, the non-clinical isolate (kp10) along with clinical isolate (kp106), were found to carry genes associated with yersiniabactin (*irp1, irp2, ybtA, ybtE, ybtP, ybtQ, ybtS, ybtT, ybtU,* and *ybtX*). Remarkably, these isolates demonstrated the highest abundance of virulence genes, as illustrated in Fig. 2.

All sequenced isolates were found to possess genes associated with various virulence factors, including *wcaG, wza, sugE,* and *treC* for capsular polysaccharide (CPS), *pgaA, pgaB, pgaC*, and *bcsA* for adhesion, and *luxS* for quorum sensing. Additionally, genes related to pilli (*ecpA*), fimbriae type I (*fimABCDEFGHIK*), and type III (*mrk ABCDFHIJ*), crucial for biofilm formation, were identified in all analyzed isolates. Multiple variations were observed in the *treC* and *wza* genes, crucial for biofilm formation, as well as in genes related to the secretion system across all isolates. Notably, significant variations were identified in genes associated with the type six secretion system (T6SS). Virulence genes for allantoin uptake were absent in all the sequenced genomes.

### Mobilome of *K. pneumonia* isolates

**Fig. 3:**
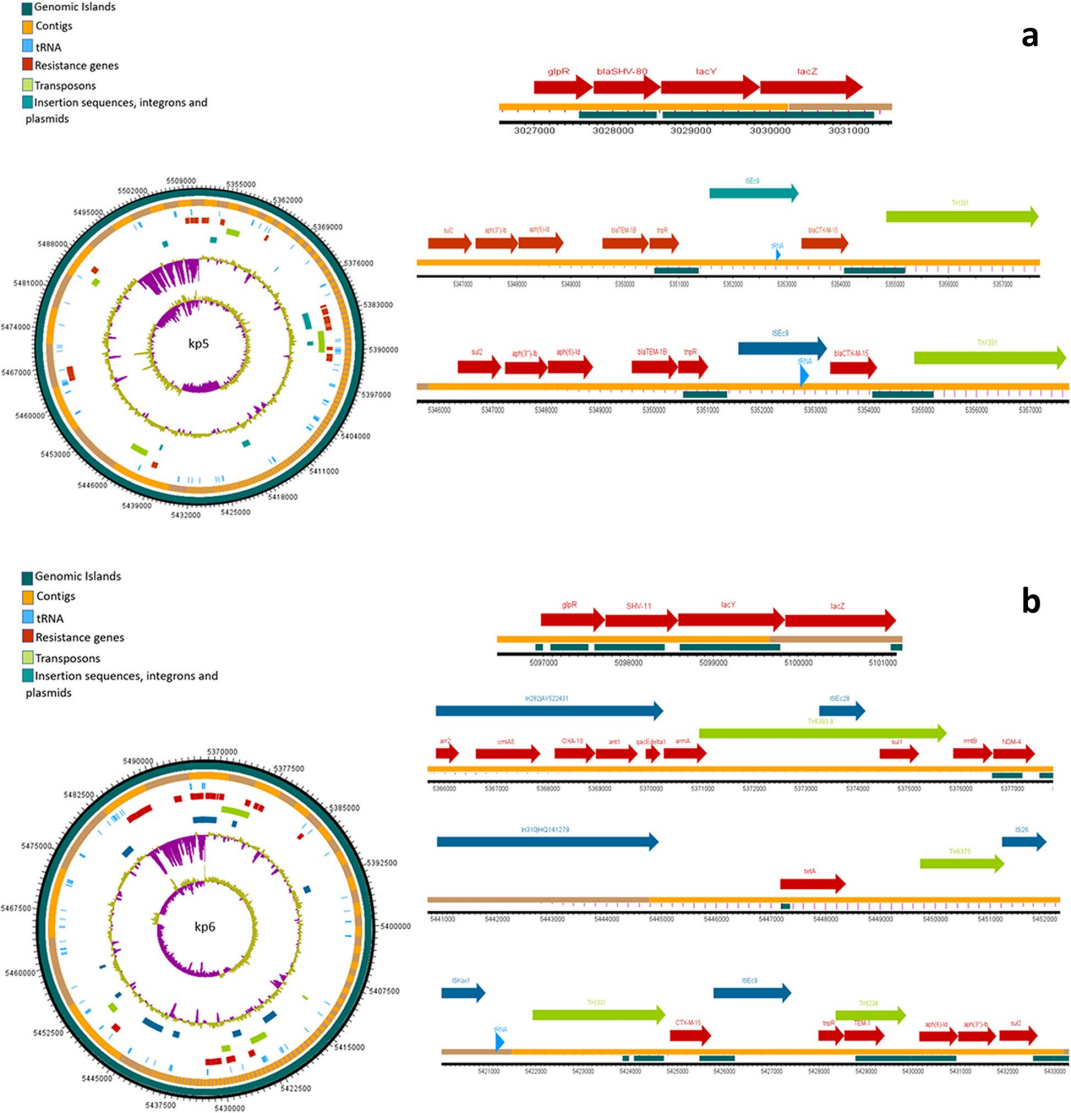
Graphical representation of the genetic environment of the resistance genes and mobile genetic elements of kp5 (**a**) and kp6 (**b**). The circular visualization of the whole genome sequence represents all the resistance genes and the MGEs. The horizontal representation shows the genetic environment of the resistance genes with specific importance to beta-lactamase genes and MGEs. The genetic environment of chromosomally encoded *bla*_*SHV*_ was found consistent in all the genomes. The *bla*_*CTM-15*_ and *bla*_*TEM*_ genes were mostly found on Tn3 transposons and ISEc9

**Fig. 4:**
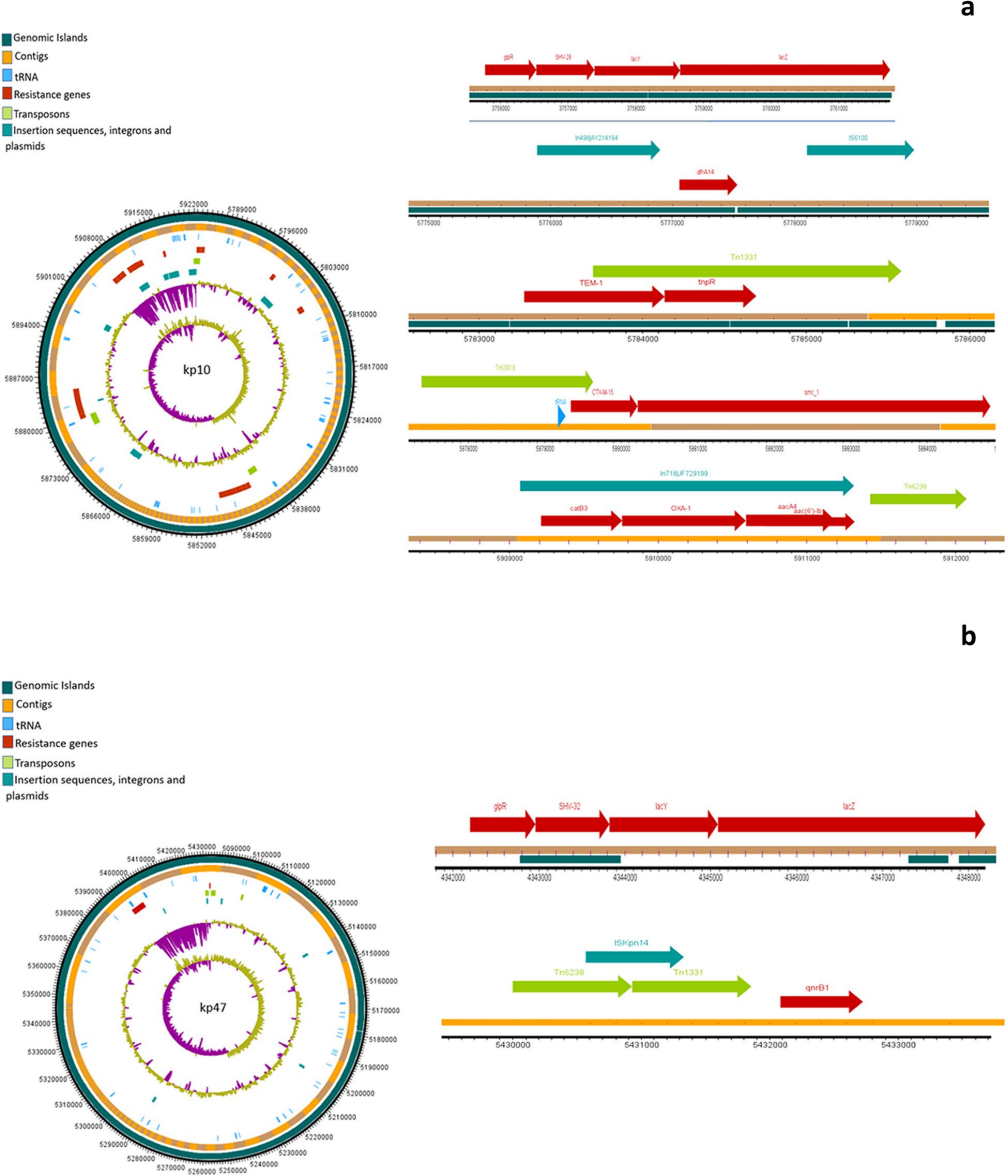
Graphical representation of the genetic environment of the resistance genes and mobile genetic elements of kp10 (**a**) and kp47. The circular visualization of the whole genome sequence represents all the resistance genes and the MGEs. The horizontal representation shows the genetic environment of the resistance genes with specific importance to beta-lactamase genes and MGEs. The genetic environment of chromosomally encoded *bla*_*SHV*_ was found consistent in all the genomes. The *bla*_*CTM-15*_ and *bla*_*TEM*_ genes were mostly found on Tn3 transposons and ISEc9

**Fig. 5:**
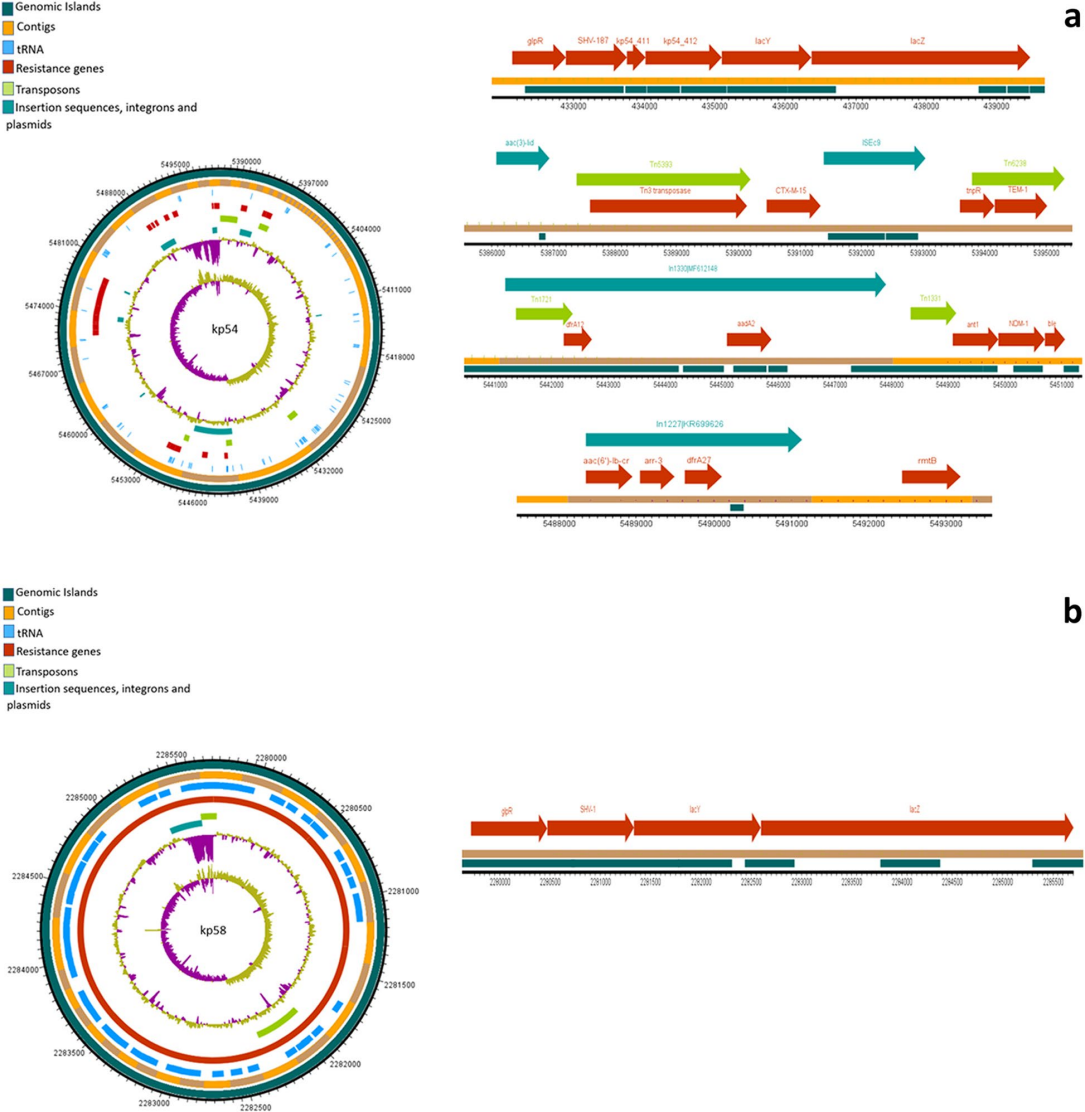
Graphical representation of the genetic environment of the resistance genes and mobile genetic elements of kp54 (**a**) and kp58. The circular visualization of the whole genome sequence represents all the resistance genes and the MGEs. The horizontal representation shows the genetic environment of the resistance genes with specific importance to beta-lactamase genes and MGEs. The genetic environment of chromosomally encoded *bla*_*SHV*_ was found consistent in all the genomes. The *bla*_*CTM-15*_ and *bla*_*TEM*_ genes were mostly found on Tn3 transposons and ISEc9

**Fig. 6:**
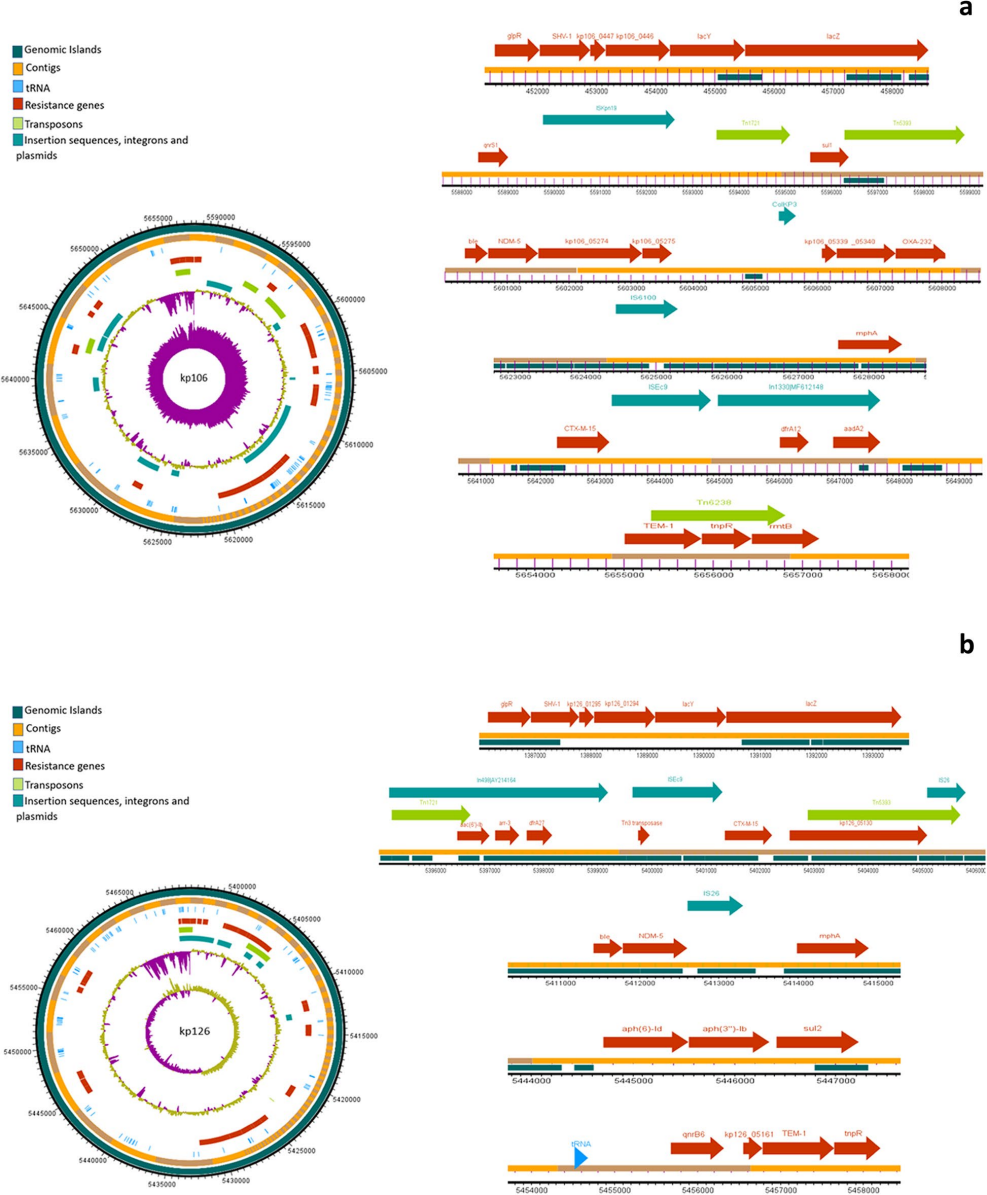
Graphical representation of the genetic environment of the resistance genes and mobile genetic elements of kp106 (**a**) and kp126. The circular visualization of the whole genome sequence represents all the resistance genes and the MGEs. The horizontal representation shows the genetic environment of the resistance genes with specific importance to beta-lactamase genes and MGEs. The genetic environment of chromosomally encoded *bla*_*SHV*_ was found consistent in all the genomes. The *bla*_*CTM-15*_ and *bla*_*TEM*_ genes were mostly found on Tn3 transposons and ISEc9

**Fig. 7:**
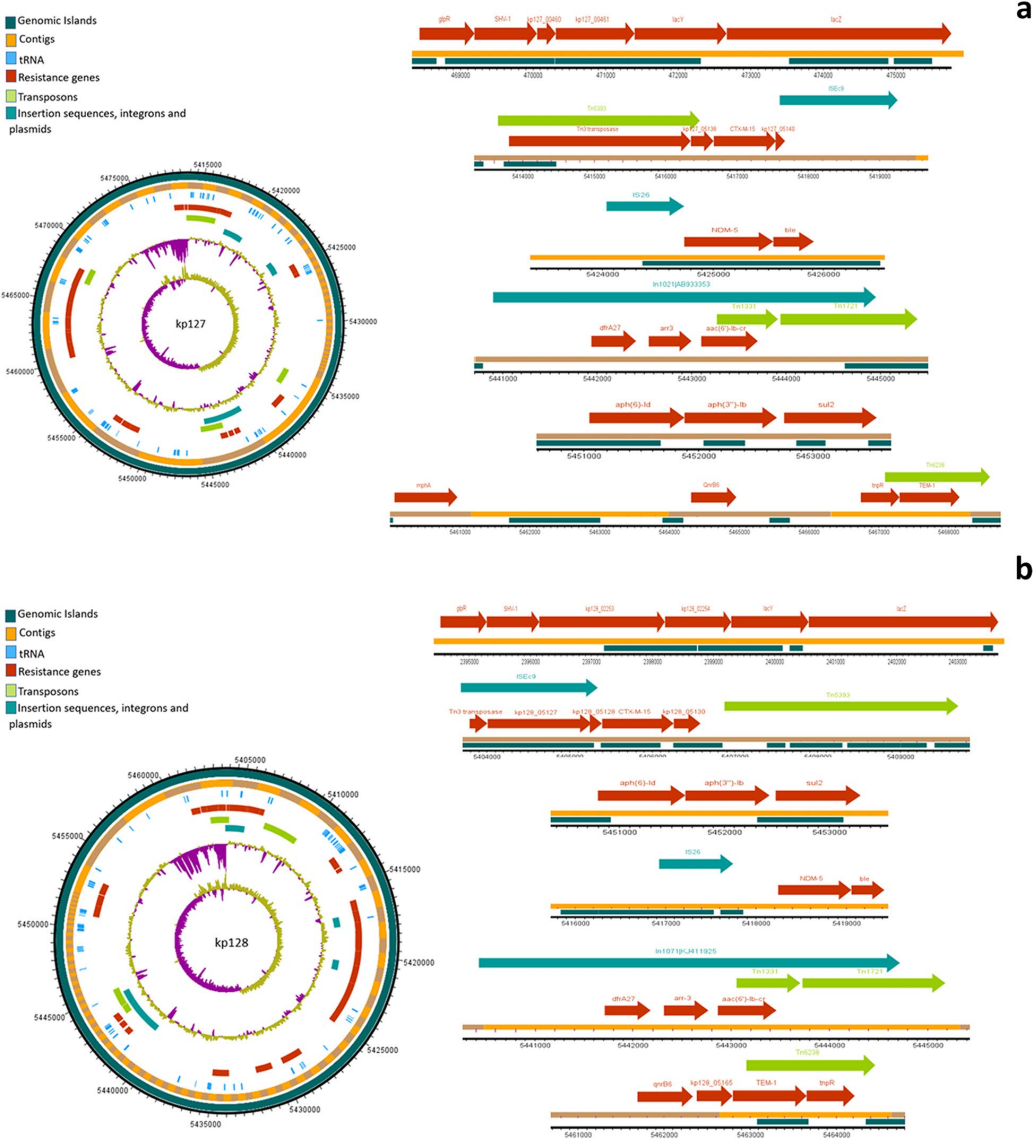
Graphical representation of the genetic environment of the resistance genes and mobile genetic elements of kp127 (**a**) and kp128. The circular visualization of the whole genome sequence represents all the resistance genes and the MGEs. The horizontal representation shows the genetic environment of the resistance genes with specific importance to beta-lactamase genes and MGEs. The genetic environment of chromosomally encoded *bla*_*SHV*_ was found consistent in all the genomes. The *bla*_*CTM-15*_ and *bla*_*TEM*_ genes were mostly found on Tn3 transposons and ISEc9

The analysis of mobile genetic elements (MGEs) revealed a consistent presence of nearly identical transposons and insertion elements in all isolates. The water isolate kp10 exhibited extensive mobilome in close proximity of the ARGs. The chromosomal encoding of all *bla*_*SHV*_ variants was notable, with insertion into the lac operon, forming a *glpR-bla*_*SHV*_*-lacY-lacZ* structure. The examination MGEs revealed the presence of nearly identical transposons and insertion elements in all isolates. Furthermore, the coexistence of *bla*_*CTX-M-15*_ and *bla*_*TEM*_, flanked by transposases/resolvase or tRNA, was observed in all isolates. Predominant mobile genetic elements, ISEc9 and Tn1331, were closely associated with both *bla*_*CTX-M-15*_ and *bla*_*TEM*_ genes. Notably, isolate kp6 exhibited additional MGEs such as Tn5393 and Tn6328. The *bla*_*OXA*_ variants appeared to be present as gene cassettes carried by class I integrons. In 5/10 isolates, *bla*_*NDM*-1_ and *bla*_*NDM*-5_ genes were frequently found adjacent to the bleomycin resistance gene *ble*, while 2/10 isolates demonstrated a co-occurrence of the *rmtB* gene (encoding 16S rRNA methylase) and *bla*_*NDM-4*_. Figures 3, 4, 5, 6 and 7 illustrate the genetic environment of the resistance genes and MGEs in the sequenced isolates.

PlasmidFinder identified at least one plasmid replicon in all of the sequenced isolates from our study. Notably, isolates kp54 and kp106 were found to harbor three distinct plasmid replicons. Among the identified plasmid replicons, various incompatibility (Inc) types were observed, such as IncH, IncF, and IncR. IncC plasmid replicon was exclusively identified in kp10. Specifically, isolates kp54, kp58, and kp106 were found to contain Col plasmids, while isolate kp47 had only the repB plasmid replicon. In addition to plasmid replicons, class I integrons were present in all the isolates. The most frequent gene cassettes observed within the class I integrons were *dfrA* and *arr*, indicating the presence of genes associated with resistance to trimethoprim (*dfrA*) and rifampicin (*arr*), respectively. Among all the *K. pneumoniae* isolates in our study, the most prevalent type of trimethoprim resistance gene was *dfrA* type, which was found in 8/10 (80%) isolates. Further analysis of the sequenced isolates revealed that five of them contained integrons carrying the *dfrA-27* gene, three isolates had integrons with *dfrA-14* trimethoprim resistance gene.

MGE Finder and IS finder revealed the presence of multiple insertion sequence (IS) elements in all of the sequenced isolates in our study (Supplementary Table 3). Among these IS elements, ISEc9 was the most commonly identified IS element, and it was consistently found in the genetic context surrounding the beta-lactamase genes. Moreover, several intact (score > 90), questionable (score 70–90), and incomplete prophage sequences (score < 70) (Supplementary Table 1) were identified in all genomes by employing the PHASTER (Phage Search Tool Enhanced Release) tool. Except genomes kp5 and kp6, all the other genomes analyzed in our study contained at least one intact prophage sequence. Among the identified prophage sequences, the Klebsi_ST16_OXA48phi5.4_NC_049450 prophage sequence was observed in isolates kp58, kp126, kp127, and kp128. Additionally, isolates kp47 and kp106 harbored the Entero_mEp235_NC_019708 prophage sequence, which is approximately 46.3 kilobases (kb) in size. Furthermore, the Entero_mEp237_NC_019704 prophage sequence, measuring approximately 57.3 kb, was identified in isolates kp126, kp127, and kp128.

### Mutations in antibiotic resistance associated genes

**Fig. 8:**
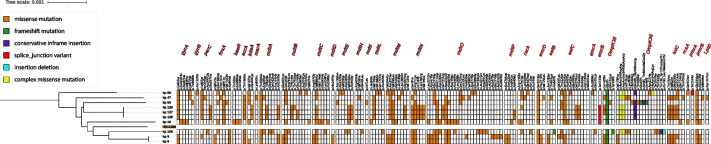
Heatmap showing the Single Nucleotide Polymorphism (SNP) between the isolates obtained in this study. Different colors correspond to missense, insertion, frameshift, and complex variations. The maximum number of variations were observed in RND efflux pump gene *mdtO*. The isolates kp47 and kp58 that had the highest number of mutations

To assess the prevalence and consequences of non-synonymous mutations in genes indirectly associated with antibiotic resistance, we performed Single Nucleotide Polymorphism (SNP) calling on the entire genome sequences using Snippy (Supplementary Table 5). The highest number of variations in genes encoding antibiotic efflux, antibiotic inactivation, antibiotic target alteration, outer membrane proteins, and lipid modification were observed in the XDR strains kp128 (*n* = 52). The water isolate kp10 also presented a significant number of non-synonymous mutations (*n* = 43) in various genes similar to other clinical isolates. All the isolates harbored genetic determinants with variations in the Type 6 Secretion System (T6SSI-III) which is an important virulence factor [23]. Remarkably the isolates kp47 and kp58 that had the lowest resistance gene frequency were among the genomes that had the significant number of mutations. Most of the mutations were observed in antibiotic efflux pump genes i.e. *mdtC, mdtM, eefC* with the highest number of snps (*n* = 63) in the *mdtO* gene. Several variations were found in genes encoding for significant outer membrane proteins OmpK35*,* Ompk36, and *tolC.* Amongst the observed variation Ile140Thr, Thr258Ser, and p.Gly3fs were the main mutations in Ompk35*.* Several types of mutations were found in the genes encoding the Ompk36 porin protein, including insertions, deletions, and missense mutations. Furthermore, all the isolates were observed to possess at least one mutation in the *pmrB* gene, *PmrAB* (two-component regulatory system), which is known to be one of the primary mechanisms for the development of resistance towards polymyxin. Furthermore, multiple mutations were identified in genes linked to fluoroquinolone resistance, with Ile83Ser being the predominant mutation in the *gyrA* gene and Ile80Ser in the *parC* gene. A heatmap was created to visually represent all non-synonymous mutations identified in the selected genes (Fig. 8).

### Mutations in porins OmpK35 and OmpK36 and their association with Carbapenem resistance

Mutations in porins, specifically OmpK35 and OmpK36, have been linked to greater resistance to carbapenemase [12, 13]. The presence of frameshift variant c.5dupG p.Gly3fs in OmpK35 was found in all our sequenced isolates. This leads to change in the amino acid sequence, causing the replacement of glycine (Gly) at position 3 with a different amino acid, and the subsequent formation of a truncated protein. Several mutations that alter the amino acid sequence of OmpK36 were detected, and one of these mutations, Thr258Ser, was found to have an impact on the stability of protein structure.

### Prediction of functional & structural consequences of SNPs in OmpK36

Using the query sequence of Ompk36 proteins, SIFT conducted a sequence search with default parameters, and the median score selected was 3.00.The mutation at position serine 258 in Ompk36 was predicted to decrease protein stability when mutated. The prediction results from SIFT and MUpro are summarized in a Table 5.

**Table 5:** Functional & structural consequences of SNPs

Protein	Mutation	SIFT		MUpro		
		Score	Prediction	▲G	Confidence Score	Prediction
**Ompk36**	Val178Pro	0.62	Tolerated	−1.295	−1	Decreases Stability
	Thr258Ser	**0.04**	Affect Protein Function	−1.048	**0.97**	Decreases Stability
	Leu307Ile	0.55	Tolerated	−0.389	−1	Decreases Stability
	Ile315Leu	1.00	Tolerated	−0.488	−1	Decreases Stability
	Asp344Glu	0.43	Tolerated	−0.809	−0.5	Decreases Stability
	His349Arg	0.56	Tolerated	−0.879	0.82	Decreases Stability

Additionally, HOPE was used to explain the effect of mutations on protein function and structure. Ompk36 is a trimeric protein made up of 16-stranded β barrels that form three separate pores. The protein has long loops connecting the β strands on the outer side and short turns on the periplasmic side. Loop 3 plays a crucial role in narrowing the pore and is folded into the barrel. Mutations in these loops are considered critical for the protein’s proper functioning [24]. A mutation in OmpK36 at position 258, where threonine is replaced by serine, affects the hydrogen bond formation with Glutamic Acid at position 282. Additionally, the mutant residue is smaller than the wild-type residue, resulting in an incorrect position to form the same hydrogen bond. Consequently, external interactions may be lost due to the mutation.

**Fig. 9:**
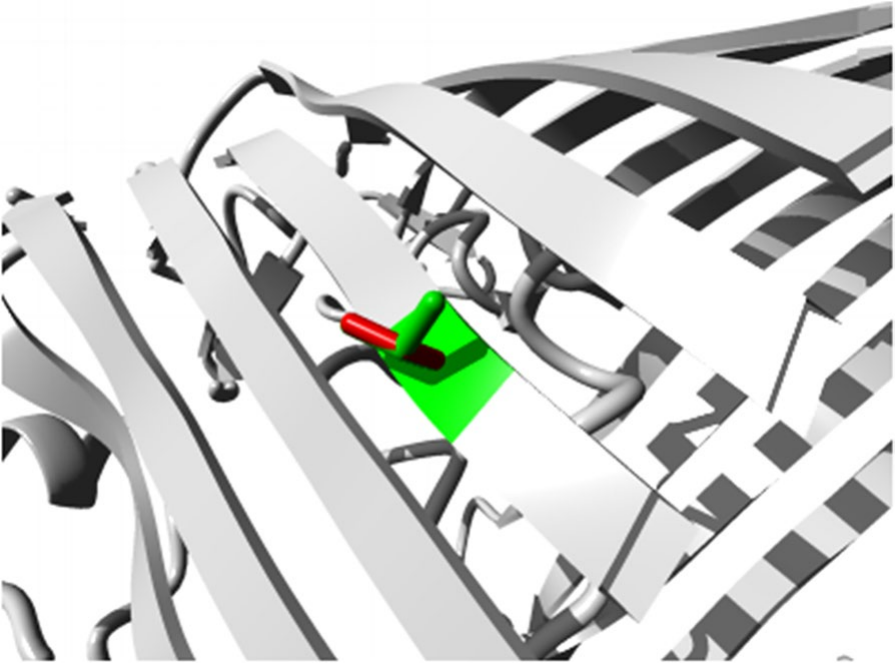
Close-up of the mutation in the OmpK36 protein. The protein is colored grey, the side chains of both the wild-type and the mutant residue are shown and colored green and red respectively

Through the 3D structure analysis of the protein, it was observed that the wild-type residue is located within a β-strand, which is its preferred secondary structure. However, the mutant residue is not located in the same secondary structure, leading to a slight destabilization of the local conformation (Supplementary Fig. 2). Furthermore, this mutation creates a void space in the protein’s core, as shown in the Fig. 9.

### Phylogenomic analysis

**Fig. 10:**
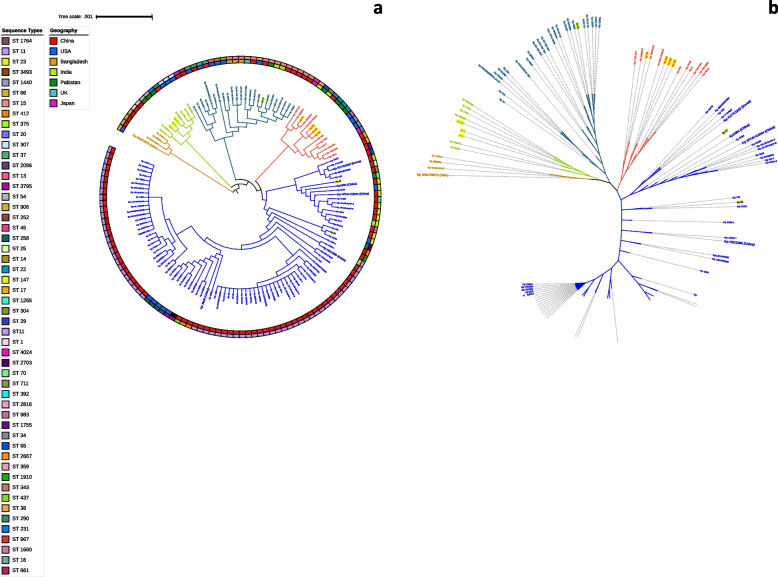
**A** Phylogenetic analysis demonstrating the diversity of *Klebsiella pneumoniae* isolates from clinical and water samples. Midpoint-rooted circular representation of the phylogenetic tree, with branch colors representing the same clades. Outer ring is annotated with the sequence types (STs) while the inner ring represents the country from where the isolates were reported. The scale bar in the phylogenetic tree represents the number of substitutions per site, indicating the genetic distance between different strains. The study strains are highlighted in yellow to distinguish from the reference strains. The reference strains, which are widely recognized and extensively studied, are indicated in bold font. **B** Un-rooted approximate-maximum likelihood phylogenetic tree constructed based on a core-genome SNPs based alignment of the sequenced isolates from this study and reference genomes. The tree was refined and annotated using iTOL (https://itol.embl.de/)

The phylogenomic analysis demonstrated substantial phylogenetic diversity among the sequenced *K. pneumoniae* isolates, as depicted in Fig. 10. Employing a core genome SNP-based approach, the analysis categorized global representative strains into five phylogroups. The sequenced genomes from our study were found to cluster within four of these five phylogroups. In the clustering process, it was noted that the neonatal strain kp5 and adult strains kp6 and kp106 formed Group II, alongside strains from China and the USA. The water isolate kp10 was clustered in Group III, along with strains from China and India. Additionally, isolates kp54, kp126, kp127, and kp128 clustered in Group IV, aligning with isolates of Indian origin. Notably, two isolates, kp47 and kp58, were positioned in the largest phylogroup, Group V, alongside global reference strains (Kp1084 and NTUH-K2044). The neonatal strain kp47 clustered with global reference strains KCTC2242 and kp1084, originating from Korea and China, respectively. On a global scale, the sequence types (STs) clustered together with either identical or closely related STs from China, USA, and India, suggesting a shared genetic lineage among geographically diverse strains.

## Discussion

The global prevalence of MDR/XDR *K. pneumoniae* has been consistently on the rise. In Pakistan, particularly, there has been a notable surge in the incidence of MDR/XDR *K. pneumoniae*, estimated at approximately 40%. This underscores the increasing concern regarding antibiotic-resistant *K. pneumoniae* infections in our country. Remarkably, the emergence of carbapenem-resistant *K. pneumoniae* (CRKP) has witnessed a significant increase, constituting 84.16% of reported cases in Pakistan [25].. During the investigation of antibiotic resistance, it was observed that the genotypic profiles of the sequenced isolates were fully consistent with their phenotypic profiles, indicating a strong association between the presence of ARGs and the observed resistance patterns. However, two strains, namely kp47 and kp58, exhibited a disparity between their genotypic and phenotypic profiles, suggesting the presence of additional mechanisms contributing to their antibiotic resistance beyond the detected resistance genes. This bacterial species has been known to be intrinsically resistant to penicillin because of the core gene *bla*_*SHV*_ present in the chromosome [22]. All of our study isolates were resistant to ampicillin due to the presence of allelic variants of the *bla*_*SHV*_ gene in the core genome. Among these variants, *bla*_*SHV-1*_ along with *bla*_*SHV-11*_ were more prominent SHV variants [26]. The variant *bla*_*SHV-187*_ detected in one of the isolates has been recently documented in MDR *Klebsiella* strains in another study [27–29]. Furthermore, the occurrence of *bla*_*CTX-M-15,*_ *bla*_*TEM-1,*_ *and bla*_*OXA*_ genes, also attributed resistance to the beta-lactam class of antibiotics. Additionally, allelic variants of class B and D carbapenemase *bla*_*NDM*_ gene NDM-1, 4, and 5 and *bla*_*OXA*_ gene OXA 1, 10, and 232, respectively, were also detected, which have been recently reported from Pakistan isolates [30–32]. Among the plasmid-mediated quinolone resistance (PMQR) genes detected in our isolates, the most dominant gene was *oqxA*, present in 70% of the isolates, which is consistent with the findings reported by *Haeli* et al. [25]*.* This was followed by the *qnrB* gene, present in 50% of the isolates, and the *aac(6′)-Ib-cr* gene, present in 30% of the isolates. The least frequent PMQR gene observed in our study was *qnrS*, present in 20% of the isolates, which differs from previous reports [33]. Mutations in *gyrA* and *parC* have been recently reported in quinolone-resistant isolates [34, 35] that were also observed in our study, here the most frequent mutation was lle83Ser (70%) in *gyrA* and lle80Ser (60%) in *parC* gene. In our study, the most common aminoglycoside resistance genes were *aac(6′)-Ib-cr, aph(3″)-Ib, aph(6)-Id, aac(3)-Iid* while *armA, aad2,* and *rmtB* were detected in two isolates, contributing High Level Aminoglycoside Resistance (HLAR) as reported by *Zhang* et al. [33]*.* Resistance genes like *LptD* and *msrE,* involved in LPS transport in ABC Transporter efflux system conferring resistance to carbapenems, rifamycin, and peptide antibiotics, have been reported elsewhere in isolates from the adult population [36, 37] were also identified in our neonatal strains. In our study samples, the biocide resistance gene *qacEdelta1*, commonly found in Gram-negative bacteria, was prevalent. This gene is associated with conferring resistance to aminoglycosides, trimethoprim, and sulfonamides through a multidrug efflux mechanism. Its presence suggests the potential for multidrug resistance development in the studied isolates, further highlighting the importance of understanding and monitoring the spread of biocide resistance genes in bacterial populations [38].

Occurrence of the *bla*_*CTX-M-15*_*, bla*_*TEM*_*, bla*_*OXA*_*, and bla*_*SHV*_ ESBL genes in similar genetic contexts in our isolates and previous reports [11] indicates that antibiotic resistance is facilitated by either intrinsic or acquired genes found on the chromosome or mobile genetic elements (MGEs) [39]. Previous studies have highlighted the significant role of certain plasmids, such as IncF plasmids, and insertion sequence ISEc9, in enabling the widespread dissemination of genes like *bla*_*CTX-M-15*_, *aac(6*′*)Ib-cr, bla*_*OXA-10*_ *and bla*_*TEM*_ through vertical gene transfer processes [22]. The study isolates kp54 and kp106, harbored globally prevailing IncF plasmids replicons that have been previously reported in *Enterobacteriaceae* often carrying virulence genes, and their role in HGT has already been confirmed [1, 11]. The IncC plasmid replicons that were identified in water isolate kp10 are particularly important as they harbor genes conferring resistance to antibiotic classes like chloramphenicol, sulfonamide, aminoglycoside, trimethoprim and AmpC β lactamase [1]. The globally distributed plasmid IncH1B(pNDM) was identified in two isolates, namely kp5, and kp6, which have previously been reported in Morocco, the United States, and the United Kingdom [1]. The strain kp47 exhibited a plasmid repB replicon that falls under the IncFIB family. This replicon is recognized for dynamic nature and plays a crucial role in facilitating the spread of antimicrobial resistance within *Klebsiella* populations [40].. The presence of a wide variety of plasmid replicons carrying genetic elements associated with both resistance and virulence factors in our isolated strains is particularly noteworthy.

The identified integrons in our study exhibited a wide variety of gene cassettes, with particular emphasis on *aadA* and *dfrA* gene cassettes. These specific gene cassettes have been previously reported to play a significant part in the global spread of these mobile genetic elements [11].. In our study, it was observed that all the isolated strains contained Class I integrons, which have been extensively researched and are recognized for their significant contribution to the global spread of resistance genes. This is primarily due to their association with various mobile elements, further enhancing their ability to disseminate ARGs widely [41]. In two of our study isolates, we identified Integron 310, which has been previously reported to capture *arr-2* and *cmIA5* in *Acinetobacter baumannii* strains in South and East Asia as well as Portugal [42, 43]. The presence of Class I integrons in natural environments that have undergone evolutionary recombination events is well-documented, further emphasizing their importance in the acquisition and spread of antibiotic-resistance genes [42, 43]. The presence of two integrons In498 and In718 carrying *dfrA12* and *catB* genes, respectively, in water isolate kp10 also supports the role and spread of integrons in pathogenic bacteria. All the isolates also had composite and unit transposons particularly Tn5393 along with a wide range of insertion sequences (IS). The presence of ISEc9 in the vicinity of beta-lactamase genes suggests its potential involvement in the spread or mobilization of these resistance genes within the bacterial population. The transposons identified in our study were classified as members of the Tn3 family of replicative transposons. This group of transposons is widely distributed and known to play a significant role in the dissemination of antibiotic resistance [44]. The high diversity of transposons and insertion sequences can be attributed to the plasticity of genomes [11].

Based upon the diverse polysaccharide components with various K and O antigens, *K. pneumoniae* has been divided into 79 capsular types amongst which eight types have been described as markers for hypervirulent strains [45]. In our study, we observed a diverse range of sequence and capsular types among the isolates that belonged to different clonal groups consistent with previous findings. The capsular type K54 is well-known for its association with hypervirulence in *Klebsiella pneumoniae* strains. Specifically, it has been linked to sequence type ST29, which is recognized as a representative hypervirulent strain of *K. pneumoniae* belonging to CG29 [26, 46]. These findings highlight the diverse genetic characteristics and global distribution of different capsular types and sequence types in *K. pneumoniae*, underscoring the significance of clonal groups and their association with antimicrobial resistance and virulence traits. The major virulence factors implicated in the pathogenicity and propensity to cause *Klebsiella pneumoniae* infections are capsule, lipopolysaccharides, fimbriae, and siderophores [3]. *RcsAB*, a two-component regulatory system that is involved in high capsule productivity [23], was identified in all the isolates. One of our study isolates had capsular type K54 one of the biomarkers for hvkp strains that had the least number of resistance genes but was found to be phenotypically more resistant and virulent. This can be explained as the hyper capsule serving as a physical barrier thus limiting the DNA uptake and consequently the HGT that’s why they harbor few resistance genes but are more challenging therapeutically [23]. Our isolates were found to contain Outer membrane protein A (OmpA) and outer membrane porins OmpK35 and OmpK36, known for their contribution to virulence [23]. The virulence genes for core and acquired siderophores were identified as described previously [22, 26, 47, 48]. The genes for type I fimbriae (*fimA-fimK*) and type III fimbriae (*mrkB-mrkJ*) and pili (*ecpA)* have established roles in biofilm formation and UTI [47, 49] and were present in all the isolates. In addition to fimbriae and pili, we have detected genetic elements like adhesion genes *pgaABC and bcsA, luxS* in quorum sensing, *treC* and *sugE* for capsule production, the combined effect of which have been reported crucial to produce an excellent biofilm [50, 51]. Several snps were observed in *treC* and *wza* genes in all the isolates as previously reported [52]. In order to further investigate the impact of these variants among the sequenced isolates, additional knock-out studies are necessary. The highest number of variations were observed in *mdtO* gene, involved in antibiotic efflux. Previous reports have shown that this gene facilitates antibiotic resistance and deletion of *mdtO* increases the sensitivity of the bacteria towards antibiotics [53]. The variant calling showed that Asp553Gly (90%) was the most common mutation followed by Glu612Asp (80%) which was observed in *mdtO* gene. Resistance to carbapenems in *K. pneumoniae* relies on several crucial factors, including modifications to the major outer membrane porins, OmpK35 and OmpK36. Additionally, the presence of extended-spectrum β-lactamases (ESBLs) or plasmid-encoded carbapenemases plays a significant role in conferring resistance to carbapenems in this bacterium [54]. A common finding in many studies consistent with our findings is the identification of non-functional porins resulting from frameshift mutations leading to truncated OmpK35 resulting in a shortened protein than the wild type [54–56]. The structural modifications observed in OmpK36 are caused by the insertion of amino acids into a specific region of the porin, known as loop 3 (L3). This results in a reduction in the diameter of the luminal space and restricted diffusion of substrates, such as antibiotics. The L3 insertions, are frequently observed in clinical isolates of *Klebsiella pneumoniae*. They have been identified in approximately 12.3% (192 out of 1557) of isolates in a diverse collection of publicly available genomes [13]. Due to the high prevalence of L3 insertions in carbapenemase-producing strains of *K. pneumoniae*, a study has been initiated to examine the specific implications of these mutations on the efficacy of newly developed or recently approved drugs that target resistant strains of *K. pneumoniae* [13]. There is currently limited information available from the Indian subcontinent about the frequency of mutations in porins, OmpK35 and OmpK36, as well as the prevalence of porin deletions in *K. pneumoniae* [57]. However, given the high prevalence of XDR isolates in Pakistan, it is essential to thoroughly investigate all possible resistance mechanisms. Moreover, understanding the significance of these mutations and their effects is essential for developing strategies to combat bacterial infections.

## Conclusion

In this study, we present comprehensive data from the whole genome sequences of *K. pneumoniae* strains isolated from neonatal, adults, and tap water from a tertiary care hospital in Pakistan. We observed significant genomic diversity amongst our sequenced isolates. The water isolate exhibited similar resistance determinants and the highest number of virulence genes, a finding of particular concern. Our results indicates high burden of antibiotic resistance and virulence associated genes among *K. pneumonia* bacterial isolates, which are putatively the main culprit to poor outcome of antibiotic treatment leading to high mortality rate. The presence of various MGEs in close association with the ARGs indicates their role in HGT between diverse organisms. We also found that spontaneous mutations in porin related genes specific for the active uptake of carbapenem antibiotic e.g. OmpK35 and OmpK36 lead to the loss of porin protein function, thus causing increased bacterial resistance toward carbapenems. The phylogenomic analysis revealed the close association of our isolates with strains from China, India and USA, indicating the global spread of MDR and XDR sequence types. This study shows an urgent need for epidemiological and molecular studies to develop a better understanding of antibiotic resistance dynamics and for guiding potential treatment of multi-drug resistant *K. pneumoniae* infections.

## Materials and methods

### Microbiology of bacterial isolates

We collected 215 *Klebsiella pneumoniae* isolates from a local tertiary care hospital during February 2019–February 2021 in Pakistan and classified these bacterial strains on the basis of their phenotypic antimicrobial resistance profiles. The isolates were collected from diverse age groups with the maximum number of isolates (*n* = 95) collected from neonates and infants accounting for 45% of the collection. Among 215 isolates, 64 were collected from blood, 58 from pus, 41 from urine, 12 from non-bronchoscopic lung lavage (NBL), 9 each from sputum & tips, 6 from fluids, 4 from swabs, 2 from tissue and 8 from hospital tap water.

### AST profiling & biofilm assay

The Kirby-Bauer disk diffusion method on Mueller-Hinton agar (Hardy Diagnostics) was used for antibiotic susceptibility testing (AST) following the Clinical and Laboratory Standards Institute (CLSI 2018) M100-S28 guidelines [58]. The isolates were tested against various classes of antibiotics including penicillin, aminoglycoside, fluoroquinolone, cephalosporin, carbapenem, phenicols and fosfomycin.

Detection of biofilm formation was performed by methods previously described [59]. Overnight cultures in fresh LB broth were inoculated in each well of a sterile 96-well polystyrene plate. After static incubation at 37 °C, the bacteria were stained for 20 mins with 0.5% crystal violet dye. After discarding the supernatant and washing with deionized water was performed thrice thus removing unattached cells. Later on, 95% ethanol was used for the elusion of the dye, and the optical density was determined at OD_550nm_. NTUH-K2044 strain, notorious as biofilm former, was selected as a positive control [59]. The biofilm assay was conducted in triplicates for each isolate.

### Selection of isolates for whole genome sequencing

Out of our bacterial cohort, a subset of 10 isolates was selected for whole genome sequencing (WGS) based on antimicrobial resistance profile and source by keeping the selection criteria consistent with the previous reports [36, 60–62]. The randomized selection of samples was performed from each subset of samples on the basis of host age (i.e. neonatal samples *n* = 7, Adults *n* = 2), antimicrobial susceptibility testing (AST) profile, and the source of the sample. The selected *K. pneumoniae* strains belonged to diverse sample types e.g. blood (*n* = 7), pus (*n* = 1), urine (*n* = 1), and hospital water (*n* = 1). Only meropenem resistant sample (*n* = 1) was selected from tap water source for sequencing. The selection of the water isolate was undertaken with the primary objectives of elucidating the reservoirs harboring resistance genes, comprehending the dynamics of pathogen transmission, and conducting an in-depth characterization of genetic diversity. The strains were investigated due to their phenotypic resistance to major classes of antibiotics and variable biofilm formatting ability.

### Genomic DNA preparation, sequencing, and assembly

The samples were first inoculated onto blood agar and incubated overnight. Subsequently, morphologically distinct colonies were streaked on MacConkey agar and incubated at 37 °C.The genomic DNA of the strains was isolated by using GeneJet Genomic DNA Purification Kit (Thermo Scientific). The concentration of the isolated genomic DNA was measured using a Nanodrop2000 UV-Vis Spectrophotometer (Thermo Scientific). Agarose gel electrophoresis with 1% (w/v) agarose gel was performed to evaluate DNA integrity. Nextra XT Library Prep Kit (Illumina, San Diego, USA) was used for preparing genomic DNA libraries [63]. Hamilton Microlab STAR automated liquid handling system was used for DNA quantification and library preparation. Kapa Biosystems Library Quantification Kit for Illumina on a Roche light cycler 96 qPCR machine was used for the quantification of the pooled libraries. Sequencing of the libraries was done on the Illumina HiSeq using a 250 bp paired-end reads protocol. During the downstream processing of raw reads, Trimmomatic v0.30 was used to trim the adapter sequences from the reads [64]. De-novo assembly was performed using SPAdes v3.7 [65] and annotation of contigs was done by Prokka v1.11 [66].

### In-silico analysis of bacterial genomes

The assembled sequences were annotated using the NCBI Prokaryotic Genome Annotation Pipeline (PGAP) (https://www.ncbi.nlm.nih.gov/genome/annotation_prok/). Comprehensive Antibiotic Resistance Database (CARD) and Resfinder were used for the prediction of antibiotic resistance genes [67, 68]. MLST 2.0 (https://cge.cbs.dtu.dk/services/MLST/) and PHYLOViZ 2.0 [69] were used to identify the sequence types and clonal groups (CG) of the isolates, respectively. BacAnt and Integron Finder were used to find out the transposons, integrons, and gene cassettes present within the genomes [67, 68]. Insertion sequences were identified using MGE and IS Finder [70, 71]. For the identification of plasmid replicons, PlasmidFinder was used [72]. Capsular typing was performed using Kaptive Web whereas Virulome was identified using VFDB (Virulence Factors Database) [73]. PHASTER (Phage Search Tool), an online server was used for the detection of intact prophage sequences [74].

### SNP calling

In addition to acquired antibiotic resistance genes, we investigated genes that play a role in antibiotic resistance, including efflux pumps, genes responsible for antibiotic inactivation, target alteration, outer membrane proteins, lipid A protein modification, and reduction in membrane permeability. Substitutions and indels (insertions and deletions) were identified by performing Single Nucleotide Polymorphism (SNP) calling using snippy 4.6.0. On the basis of literature review 43 genes were selected involved in antibiotic efflux for the detection of the variants and their putative role in contributing towards antibiotic resistance. High-quality non-synonymous SNPs were examined manually and were used for interpretation.

### Prediction of functional & structural consequences of SNPs in OmpK36

To check to functional consequences of snps in porin OmpK36, various computational tools were utilized to examine how non-synonymous SNPs (nsSNPs) affect the structure and function, and their impact on antibiotic resistance. Sorting Intolerant from Tolerant (SIFT) a program was employed to predict the phenotypic impacts of nsSNPs [75]. Similarly, the functional impacts of mutations were predicted using SNAP which employs a neural network (NN) as a machine learning technique. SNAP uses evolutionary information from multiple sequence alignments to differentiate affected variants from neutral ones. If the annotation is available, the tool also calculates structural features such as secondary structure and solvent accessibility. SNAP achieved an accuracy of 82%, as determined by cross-validation of over 100,000 experimentally annotated variants, using sustained two-state accuracy (effect/neutral) [76].

The impact of a mutation on protein 3D structure was investigated through the use of Have Our Protein Explained (HOPE), a web-based application that leverages structural information of a specific protein to predict the structural effects of a mutation [77]. To evaluate the influence of nsSNPs on protein structure stability, the web-based server MUpro was utilized https://mupro.proteomics.ics.uci.edu/ [78]. MUpro, a computational tool, predicts the effect of a single-site amino acid mutation on protein stability. It achieves an accuracy rate of 84% as determined by 20-fold cross-validation. This set of machine learning programs includes Support Vector Machines (SVM) and Neural Networks (NN) [79].

### Construction of homology-based models of mutant proteins

Homology modeling was used to predict mutant models of each SNP, by mapping the SNPs on the 3-dimensional structure of a protein. The selection of a template molecule was based on the results of the Basic Local Alignment Search Tool (BLAST) and the best *E*-value [80]. The 6RCP molecule (crystal structure of the ompk36 clinical isolate ST258 from *K. pneumonia*) was chosen as the template One hundred models were generated for each mutation using Modeller V10.4 [81]. The Discrete Optimized Protein Energy (DOPE) score is a statistical potential used in protein structure modeling to assess the quality of a given model. The DOPE score is based on statistical distributions of atom-atom interactions observed in a large set of known protein structures [82]. The top-performing models were selected based on their DOPE scores. To assess the effect of mutations on the protein’s three-dimensional structure, the mutant models were individually compared to the wild-type model, and the root-mean-square deviation (RMSD) was calculated. This allowed for quantifying the differences in spatial arrangement between the mutant and wild-type structures.

### Phylogenomic analysis

Phylogenetic analysis of the sequenced genomes was conducted alongside complete whole genomes of antibiotic-resistant *Klebsiella pneumoniae* strains obtained from different geographical locations using PATRIC (www.patric.org) and NCBI (https://ncbi.nlm.nih.gov/) available till June 2021, to obtain a current epidemiological relatedness and evolutionary analysis.

The pangenome of the sequences was analyzed using Bacterial Pangenome Analysis Pipeline (BPGA). The core-genome alignment file output generated from BPGA was used for the construction of maximum likelihood (ML) tree with FastTree v2.1.10. For visualization and annotation of phylogenetic trees Interactive tree of life (iTOL) was used [83].

### Supplementary Information

**Supplementary Material 1.**

## Data Availability

All data generated or analyzed during this study are included in this article [and its supplementary information files]. The whole genome sequences used for phylogenomic analysis are publicly available and retrieved from NCBI (https://www.ncbi.nlm.nih.gov/genome/?term=klebsiella+pneumoniae%2B). The Whole Genome Shotgun project of the individual isolate has been deposited at DDBJ/ENA/GenBank under the accession numbers and the version described in this paper is as follow.

**Table:** 

Isolate	Bioproject	Web link
kp5	PRJNA743648	https://www.ncbi.nlm.nih.gov/bioproject/?term=PRJNA743648
kp6	PRJNA744871	https://www.ncbi.nlm.nih.gov/bioproject/?term=PRJNA744871
kp10	PRJNA826469	https://www.ncbi.nlm.nih.gov/bioproject/?term=PRJNA826469
kp47	PRJNA814905	https://www.ncbi.nlm.nih.gov/bioproject/?term=PRJNA814905
kp54	PRJNA766411	https://www.ncbi.nlm.nih.gov/bioproject/?term=PRJNA766411
kp58	PRJNA817217	https://www.ncbi.nlm.nih.gov/bioproject/?term=PRJNA817217
kp106	PRJNA827029	https://www.ncbi.nlm.nih.gov/bioproject/?term=PRJNA827029
kp126	PRJNA832736	https://www.ncbi.nlm.nih.gov/bioproject/?term=PRJNA832736
kp127	PRJNA832741	https://www.ncbi.nlm.nih.gov/bioproject/?term=PRJNA832741
kp128	PRJNA833061	https://www.ncbi.nlm.nih.gov/bioproject/?term=PRJNA833061
