# Supplementary material for: Exploring the resistome, virulome, and mobilome of multidrug-resistant Klebsiella pneumoniae isolates: deciphering the molecular basis of carbapenem resistance
Source: BMC Genomics. 2024 Apr 25;25:408. doi: 10.1186/s12864-024-10139-y (PMC11044325; doi:10.1186/s12864-024-10139-y)
Supplement: Supplementary file 1 — Supplementary Material 1. [file 12864_2024_10139_MOESM1_ESM.docx]

**
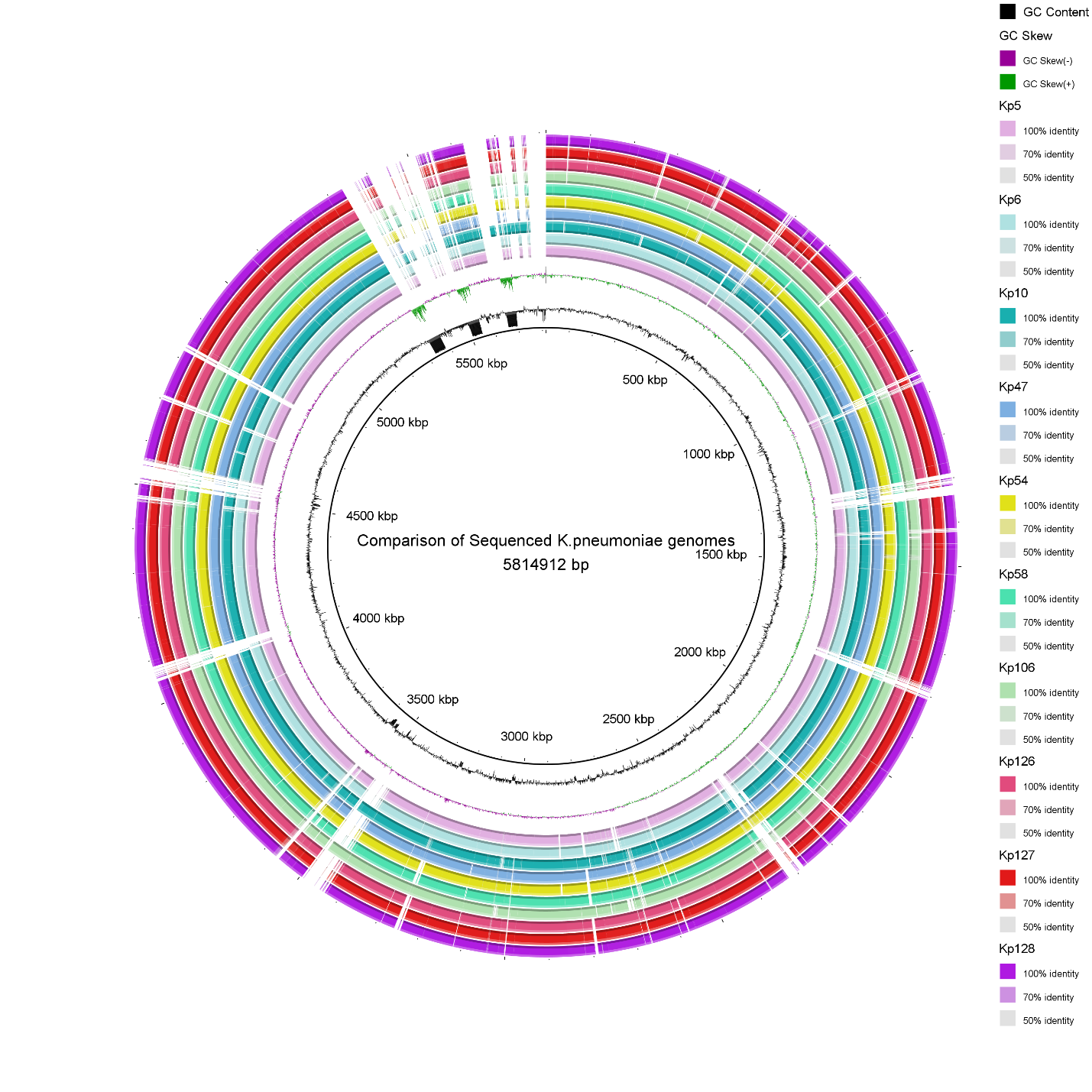
**

**Supplementary Figure 1: Comparative genomic map of 10 draft genomes of *K*. *pneumoniae* against reference genome HS11286 exhibiting number of variations. The black histogram represents the GC content, and the green-purple histogram represents the GC skew. Each ring represents WGS of single *K. pneumoniae*, shown in different colors. The outermost ring indicates the genomic position of the reference genome (HS11286). Positions covered by BLASTN alignments are indicated in solid colors and gaps (white spaces) represent genomic regions not covered by BLASTN alignments.**

**
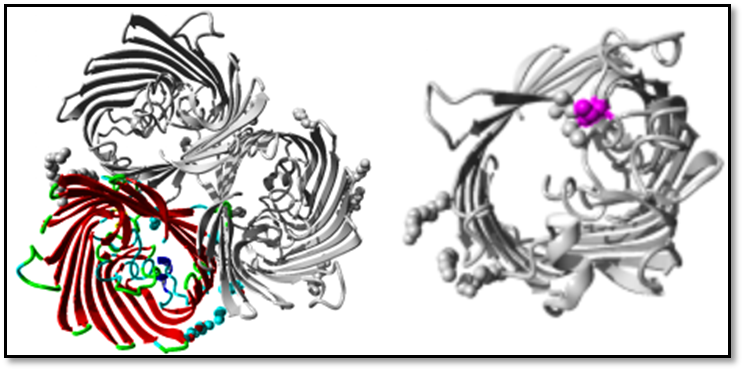
**

**Supplementary Figure 2*:* An overview of the OmpK36 protein shown in a ribbon presentation, where different elements are color-coded. α-helices are shown in blue, β-strands in red, turns in green, helices in yellow, and random coils in cyan. On the right side of the figure, the protein is colored grey, and the side chain of the mutated residue (Thr258Ser) is depicted in magenta and represented as small balls.**

**Supplementary Table 1 :** The Whole Genome Shotgun project of the individual isolate deposited at DDBJ/ENA/GenBank under the accession numbers and the version described in this paper.

| Isolate | Bioproject | Biosample | Accession Number | Version |
| --- | --- | --- | --- | --- |
| kp5 | PRJNA743648 | SAMN20056937 | JAHUTK000000000 | JAHUTK010000000 |
| kp6 | PRJNA744871 | SAMN20119844 | JAHWGG000000000 | JAHWGG010000000 |
| kp10 | PRJNA826469 | SAMN27566613 | JALOCO000000000 | JALOCO010000000 |
| kp47 | PRJNA814905 | SAMN26578426 | JALGBP000000000 | JALGBP010000000 |
| kp54 | PRJNA766411 | SAMN21844678 | JAIWJC000000000 | JAIWJC010000000 |
| kp58 | PRJNA817217 | SAMN26750226 | JALGBQ000000000 | JALGBQ010000000 |
| kp106 | PRJNA827029 | SAMN27594539 | JALOCO000000000 | JALOCO010000000 |
| kp126 | PRJNA832736 | SAMN27917750 | JAMBZI000000000 | JAMBZI010000000 |
| kp127 | PRJNA832741 | SAMN27917783 | JAMBZJ000000000 | JAMBZJ010000000 |
| kp128 | PRJNA833061 | SAMN27957732 | JALRLZ000000000 | JALRLZ010000000 |

**SupplementaryTable 2. Genomic features and statistics of sequenced *K. pneumoniae* strains**

| Strains | kp5 | kp6 | kp10 | kp47 | kp54 | kp58 | kp106 | kp126 | kp127 | kp128 |
| --- | --- | --- | --- | --- | --- | --- | --- | --- | --- | --- |
| Genome size (Mbp) | 5.54 | 5.54 | 5.96 | 5.65 | 5.50 | 5.45 | 5.67 | 5.48 | 5.48 | 5.49 |
| G+C Content (%) | 57.14 | 57.15 | 56.77 | 57.02 | 57.16 | 57.37 | 56.93 | 57.27 | 57.27 | 57.27 |
| # Contigs | 85 | 76 | 161 | 102 | 46 | 79 | 71 | 73 | 56 | 67 |
| Largest contig | 440056 | 657567 | 412946 | 463226 | 1814165 | 607008 | 899761 | 1856990 | 1856835 | 1170222 |
| N50 | 214505 | 259403 | 151712 | 229233 | 479061 | 278874 | 338602 | 294149 | 529048 | 529048 |
| N75 | 106512 | 259403 | 58437 | 108848 | 479061 | 153134 | 338602 | 294149 | 193280 | 193280 |
| CDS | 1874 | 5558 | 6052 | 3880 | 5450 | 5450 | 5723 | 5456 | 5450 | 5476 |
| tRNA | 32 | 87 | 83 | 53 | 85 | 85 | 80 | 84 | 84 | 84 |
| mRNA | 5 | 14 | 19 | 7 | 15 | 15 | 16 | 15 | 15 | 15 |
| Accessory Genes | 2580 | 2581 | 2886 | 2670 | 2550 | 2464 | 2658 | 2523 | 2527 | 2525 |

**Supplementary Table 3:** Phage sequences identified in sequenced isolates

| Sr.no | Genome | Region Length | Completeness | Most Common phage |
| --- | --- | --- | --- | --- |
| 1 | **kp5** | 31.8Kb | incomplete | PHAGE_Entero_mEp237_NC_019704 |
|  |  | 11.3Kb | incomplete | PHAGE_Entero_P4_NC_001609 |
|  |  | 28.9Kb | questionable | PHAGE_Escher_HK639_NC_016158 |
|  |  | 15.6Kb | questionable | PHAGE_Stx2_c_Stx2a_F451_NC_049924 |
|  |  | 17.3Kb | questionable | PHAGE_Salmon_SJ46_NC_031129 |
|  |  | 7.9Kb | incomplete | PHAGE_Stx2_c_1717_NC_011357 |
|  |  | 10.9Kb | incomplete | PHAGE_Salmon_Fels_1_NC_010391 |
|  |  | 3.3Kb | incomplete | PHAGE_Stx2_c_Stx2a_F451_NC_049924 |
| 2 | **kp127** | 35.4Kb | intact | PHAGE_Klebsi_ST16_OXA48phi5.4_NC_049450 |
|  |  | 57.3Kb | intact | PHAGE_Entero_mEp237_NC_019704 |
|  |  | 33.7Kb | incomplete | PHAGE_Escher_TL_2011b_NC_019445 |
|  |  | 25Kb | questionable | PHAGE_Escher_500465_1_NC_049342 |
|  |  | 12.6Kb | incomplete | PHAGE_Escher_RCS47_NC_042128 |
|  |  | 8.9Kb | incomplete | PHAGE_Entero_P1_NC_005856 |
|  |  | 9.3Kb | incomplete | PHAGE_Salmon_SJ46_NC_031129 |
| 3 | **kp47** | 28.1Kb | incomplete | PHAGE_Escher_HK639_NC_016158 |
|  |  | 46.3Kb | intact | PHAGE_Entero_mEp235_NC_019708 |
|  |  | 23.3Kb | intact | PHAGE_Salmon_118970_sal3_NC_031940 |
|  |  | 22.7Kb | incomplete | PHAGE_Pseudo_phi297_NC_016762 |
|  |  | 8.4Kb | incomplete | PHAGE_Escher_RCS47_NC_042128 |
|  |  | 19.1Kb | questionable | PHAGE_Escher_RCS47_NC_042128 |
|  |  | 27.5Kb | incomplete | PHAGE_Escher_500465_1_NC_049342 |
|  |  | 12Kb | incomplete | PHAGE_Salmon_SEN34_NC_028699 |
|  |  | 24.4Kb | questionable | PHAGE_Salmon_SEN34_NC_028699 |
|  |  | 7.8Kb | incomplete | PHAGE_Salmon_SEN34_NC_028699 |
|  |  | 12.3Kb | incomplete | PHAGE_Escher_TL_2011b_NC_019445 |
|  |  | 5.3Kb | incomplete | PHAGE_Salmon_SEN34_NC_028699 |
|  |  | 5.3Kb | incomplete | PHAGE_Stx2_c_1717_NC_011357 |
| 4 | **kp58** | 9.1Kb | incomplete | PHAGE_Entero_HK446_NC_019714 |
|  |  | 22.6Kb | questionable | PHAGE_Escher_500465_1_NC_049342 |
|  |  | 11.4Kb | incomplete | PHAGE_Escher_RCS47_NC_042128 |
|  |  | 37.7Kb | intact | PHAGE_Klebsi_ST16_OXA48phi5.4_NC_049450 |
|  |  | 27.5Kb | questionable | PHAGE_Entero_P4_NC_001609 |
|  |  | 23.6Kb | incomplete | PHAGE_Entero_mEp237_NC_019704 |
|  |  | 18.6Kb | incomplete | PHAGE_Klebsi_phiKO2_NC_005857 |
| 5 | **kp128** | 20.9Kb | incomplete | PHAGE_Escher_TL_2011b_NC_019445 |
|  |  | 35.3Kb | intact | PHAGE_Klebsi_ST16_OXA48phi5.4_NC_049450 |
|  |  | 57.3Kb | intact | PHAGE_Entero_mEp237_NC_019704 |
|  |  | 25Kb | questionable | PHAGE_Escher_500465_1_NC_049342 |
|  |  | 12.6Kb | incomplete | PHAGE_Entero_P1_NC_005856 |
|  |  | 8.9Kb | incomplete | PHAGE_Escher_RCS47_NC_042128 |
|  |  | 9.3Kb | incomplete | PHAGE_Salmon_SJ46_NC_031129 |
| 6 | **kp10** | 45.6Kb | questionable | PHAGE_Burkho_phiE255_NC_009237 |
|  |  | 12.7Kb | questionable | PHAGE_Escher_500465_1_NC_049342 |
|  |  | 17.5Kb | incomplete | PHAGE_Propio_PFR2_NC_031108 |
|  |  | 56.8Kb | intact | PHAGE_Phage_Gifsy_1_NC_010392 |
|  |  | 11.5Kb | incomplete | PHAGE_Escher_RCS47_NC_042128 |
|  |  | 45.9Kb | intact | PHAGE_Klebsi_phiKO2_NC_005857 |
|  |  | 16.2Kb | incomplete | PHAGE_Entero_P1_NC_005856 |
|  |  | 11.3Kb | incomplete | PHAGE_Escher_RCS47_NC_042128 |
|  |  | 10.9Kb | incomplete | PHAGE_Escher_RCS47_NC_042128 |
|  |  | 10.9Kb | incomplete | PHAGE_Escher_RCS47_NC_042128 |
| 7 | **kp6** | 22Kb | incomplete | PHAGE_Entero_P4_NC_001609 |
|  |  | 31.8Kb | incomplete | PHAGE_Entero_mEp237_NC_019704 |
|  |  | 12.6Kb | incomplete | PHAGE_Escher_RCS47_NC_042128 |
|  |  | 28.9Kb | questionable | PHAGE_Escher_HK639_NC_016158 |
|  |  | 5.4Kb | incomplete | PHAGE_Stx2_c_Stx2a_F451_NC_049924 |
|  |  | 7.9Kb | incomplete | PHAGE_Escher_SH2026Stx1_NC_049919 |
|  |  | 10.9Kb | incomplete | PHAGE_Salmon_Fels_1_NC_010391 |
|  |  | 3.3Kb | incomplete | PHAGE_Stx2_c_Stx2a_F451_NC_049924 |
| 8 | **kp54** | 5.5Kb | intact | PHAGE_Bacill_B4_NC_018863 |
|  |  | 51.7Kb | intact | PHAGE_Salmon_Fels_1_NC_010391 |
|  |  | 27.9Kb | incomplete | PHAGE_Strept_Austintatious_NC_048153 |
|  |  | 44Kb | intact | PHAGE_Salmon_SEN34_NC_028699 |
|  |  | 8.5Kb | incomplete | PHAGE_Salmon_SJ46_NC_031129 |
|  |  | 13.1Kb | incomplete | PHAGE_Entero_P1_NC_005856 |
| 9 | **kp106** | 11Kb | incomplete | PHAGE_Salmon_SEN8_NC_047753 |
|  |  | 28.8Kb | incomplete | PHAGE_Salmon_118970_sal3_NC_031940 |
|  |  | 42.8Kb | intact | PHAGE_Entero_mEp235_NC_019708 |
|  |  | 26.8Kb | questionable | PHAGE_Entero_mEp460_NC_019716 |
|  |  | 41.7Kb | incomplete | PHAGE_Entero_933W_NC_000924 |
|  |  | 18.7Kb | incomplete | PHAGE_Mycoba_Thonko_NC_051580 |
| 10 | **kp126** | 65Kb | intact | PHAGE_Entero_mEp237_NC_019704 |
|  |  | 35.1Kb | intact | PHAGE_Klebsi_ST16_OXA48phi5.4_NC_049450 |
|  |  | 33.7Kb | incomplete | PHAGE_Escher_TL_2011b_NC_019445 |
|  |  | 25Kb | questionable | PHAGE_Escher_500465_1_NC_049342 |
|  |  | 12.6Kb | incomplete | PHAGE_Salmon_SJ46_NC_031129 |
|  |  | 8.9Kb | incomplete | PHAGE_Escher_RCS47_NC_042128 |
|  |  | 9.3Kb | incomplete | PHAGE_Salmon_SJ46_NC_031129 |

**Supplementry Table S4:** Sequence types (ST), antibiotic resistance genes, integrons, insertion sequences and transposons found in the *Klebsiella pneumoniae*

| Genome Code (MLST) | Integron  (Accession Number) | Integron Class | Gene Cassette Arrays | | | Insertion Sequences (IS) | Transposons (Tn) |
| --- | --- | --- | --- | --- | --- | --- | --- |
|  |  |  | **GC1** | **GC2** | **GC3** |  |  |
| kp5  (ST54) | In310\|HQ141279 | Class I | arr-2 | cmIA5 | _ | ISEc9, ISKpn26, ISEc28, ISKox1 | Tn5393.9 , Tn6375  Tn6196, Tn1331  Tn1721.1 |
|  | In282\|AY522431 | Class I |  |  |  |  |  |
| kp6  (ST54) | In310\|HQ141279 | Class I | _ | _ | _ | ISEc9, IS26,  IS6100, ISEc28,  IS5, ISKpn21,  ISKox1, | Tn5393.9, Tn6375  Tn1331, Tn6238  Tn1721.1 |
| kp10  (ST15) | In498\|AY214164 | Class I | dfrA14 | _ | _ | ISKpn8,ISKpn33,  ISKpn19, IS6100,  ISEc9, ISKpn21,  IS5075 | Tn5393.9, Tn6375  Tn6238, Tn1331  Tn1721.1 |
|  | In718\|JF729199 | Class I | catB3 | _ | _ |  |  |
| kp47  (ST70) | _ |  | _ | _ | _ | ISKpn14, IS26,  ISKpn43,ISKpn34, IS30 | Tn5393.9, Tn6375  Tn6238,Tn1331 |
| kp54  (ST29) | In1330\|MF612148 | Class I | arr-3 | dfrA27 |  | ISEc9, IS26,  IS6100 | Tn5393.9, Tn6375  Tn1331, Tn6238  Tn1721.1 |
|  | In1227\|KR699626 | Class I | dfrA27 |  |  |  |  |
| kp58  (ST2703) | _ |  | _ | _ | _ | IS26 | Tn5393.9,Tn6375 |
| kp106  (ST231) | In1330\|MF612148 | Class I | dfrA14 |  |  | ISKpn19, IS6100,  ISEc9, ISKox1,  ISKpn25, ISKpn38, ISKpn14 | Tn5393.9,Tn6375  Tn6238, Tn1331  Tn1721.1 |
| kp126  (ST22) | _ |  | _ | _ | _ | IS26, IS6100, ISEc9 | Tn5393.9, Tn6375  Tn1331, Tn1721.1 |
| kp127  (ST22) | In1021\|AB933353 | Class I | arr-3 | aadA16 | dfrA27 | IS26, IS6100, ISEc9 | Tn5393.9, Tn6375  Tn1331, Tn1721.1 |
| kp128  (ST22) | In1071\|KJ411925 | Class I | arr-3 | aadA16 | dfrA27 | IS26, IS6100, ISEc9 | Tn5393.9, Tn6375  Tn1331, Tn1721.1 |
|  | In986\|KF386160 | Class I | aadA2 | dfrA1 |  |  |  |
|  | In431\|AY029772 | Class I | aad5 |  |  |  |  |

**Supplementary Table S5**. Non-Synonymous SNPs detected in genes related to antibiotic resistance in eleven sequenced isolates using HS11286 as reference genome.

| Locus tags | Gene | Mechanism | kp5 | kp6 | kp10 | kp47 | kp54 | kp58 | kp  106 | kp  126 | kp  127 | kp 128 | Total |
| --- | --- | --- | --- | --- | --- | --- | --- | --- | --- | --- | --- | --- | --- |
| KPHS_11890 | *acrA* | Antibiotic Efflux | 1 | 1 | 0 | 1 | 1 | 0 | 1 | 1 | 1 | 1 | 9 |
| KPHS_11880 | *acrB* |  | 0 | 0 | 0 | 0 | 0 | 0 | 0 | 0 | 0 | 0 | 0 |
| KPHS_17090 | *kdeA* |  | 0 | 1 | 1 | 1 | 1 | 1 | 0 | 1 | 1 | 1 | 9 |
| KPHS_07630 | *LptD* |  | 0 | 0 | 0 | 0 | 1 | 0 | 0 | 0 | 0 | 0 | 1 |
| KPHS_p300680 | *qacEdelta1* |  | 0 | 0 | 0 | 0 | 0 | 0 | 0 | 0 | 0 | 0 | 0 |
| KPHS_25470 | *marA* |  | 0 | 0 | 0 | 1 | 0 | 0 | 0 | 0 | 0 | 0 | 1 |
| KPHS_35860 | *mdtA* |  | 2 | 2 | 1 | 2 | 1 | 1 | 4 | 3 | 3 | 3 | 24 |
| KPHS_35870 | *mdtB* |  | 2 | 2 | 1 | 2 | 1 | 3 | 2 | 2 | 2 | 2 | 21 |
| KPHS_35880 | *mdtC* |  | 2 | 2 | 4 | 1 | 0 | 1 | 2 | 1 | 1 | 1 | 45 |
| KPHS_00130 | *mdtD* |  | 1 | 1 | 0 | 0 | 0 | 0 | 0 | 0 | 0 | 0 | 2 |
| KPHS_35890 | *mdtE* |  | 0 | 0 | 1 | 1 | 0 | 2 | 2 | 2 | 2 | 2 | 13 |
| KPHS_19430 | mdtG |  | 0 | 0 | 0 | 0 | 0 | 0 | 0 | 0 | 0 | 0 | 15 |
| KPHS_19540 | mdtH |  | 0 | 0 | 1 | 1 | 0 | 0 | 1 | 0 | 0 | 0 | 3 |
| KPHS_24750 | mdtI |  | 0 | 0 | 0 | 0 | 1 | 0 | 0 | 0 | 0 | 0 | 1 |
| KPHS_52840 | mdtL |  | 0 | 0 | 1 | 1 | 1 | 2 | 1 | 1 | 1 | 1 | 4 |
| KPHS_06130 | mdtM |  | 3 | 3 | 2 | 2 | 2 | 4 | 1 | 3 | 3 | 3 | 29 |
| KPHS_43660 | mdtN |  | 2 | 2 | 2 | 1 | 1 | 1 | 2 | 5 | 5 | 5 | 28 |
| KPHS_43670 | mdtO |  | 7 | 6 | 8 | 5 | 5 | 4 | 10 | 3 | 3 | 3 | 63 |
| KPHS_43680 | mdtP |  | 7 | 2 | 1 | 0 | 0 | 0 | 0 | 0 | 1 | 1 | 12 |
| KPHS_18030 | *rarA* |  | 0 | 0 | 2 | 2 | 0 | 2 | 1 | 1 | 1 | 0 | 11 |
| KPHS_52500 | *emrD* |  | 2 | 2 | 1 | 2 | 1 | 1 | 1 | 1 | 1 | 1 | 14 |
| KPHS_52090 | *EefB* |  | 1 | 1 | 1 | 1 | 0 | 0 | 1 | 0 | 0 | 0 | 6 |
| KPHS_52100 | *EefC* |  | 3 | 1 | 2 | 1 | 2 | 3 | 2 | 4 | 3 | 3 | 29 |
| KPHS_40890 | *emrA* |  | 0 | 0 | 0 | 0 | 0 | 1 | 1 | 0 | 0 | 0 | 2 |
| KPHS_40900 | *emrB* |  | 0 | 0 | 1 | 0 | 0 | 0 | 0 | 1 | 1 | 1 | 6 |
| KPHS_06030 | *fosA* | Antibiotic Inactivation | 2 | 2 | 2 | 1 | 3 | 2 | 2 | 1 | 1 | 1 | 19 |
| KPHS_07600 | *rsmA* |  | 0 | 0 | 0 | 0 | 0 | 0 | 0 | 0 | 0 | 0 | 0 |
| KPHS_35910 | *baeR* |  | 0 | 0 | 3 | 0 | 0 | 0 | 0 | 0 | 0 | 0 | 3 |
| KPHS_p300550 | *rmtB* | Antibiotic Target Alteration | 0 | 0 | 0 | 0 | 0 | 0 | 0 | 0 | 0 | 0 | 0 |
| KPHS_37060 | *gyrA* |  | 1 | 1 | 3 | 2 | 3 | 2 | 0 | 1 | 1 | 1 | 15 |
| KPHS_52750 | gyrB |  | 0 | 0 | 0 | 0 | 0 | 1 | 0 | 0 | 0 | 0 | 1 |
| KPHS_45640 | *parC* |  | 1 | 0 | 0 | 2 | 1 | 2 | 0 | 2 | 2 | 2 | 12 |
| KPHS_45710 | *parE* |  | 0 | 0 | 0 | 0 | 0 | 0 | 0 | 0 | 0 | 0 | 0 |
| KPHS_18650 | *OmpA* | Outer Membrane Protein | 0 | 0 | 0 | 0 | 0 | 0 | 0 | 0 | 0 | 0 | 0 |
| KPHS_18380 | *OmpK35* |  | 2 | 2 | 1 | 2 | 1 | 2 | 2 | 2 | 2 | 2 | 20 |
| KPHS_37010 | *OmpK36* |  | 1 | 1 | 0 | 4 | 6 | 4 | 8 | 3 | 3 | 3 | 0 |
| KPHS_45760 | *tolC* |  | 4 | 3 | 1 | 5 | 0 | 1 | 0 | 4 | 4 | 4 | 26 |
| KPHS_34420 | *rcsA* | Modification of lipid A | 0 | 0 | 1 | 0 | 0 | 1 | 0 | 0 | 0 | 0 | 3 |
| KPHS_37040 | *rcsB* |  | 0 | 0 | 0 | 0 | 0 | 0 | 0 | 0 | 0 | 0 | 0 |
| KPHS_20130 | *phoP* |  | 0 | 0 | 0 | 0 | 0 | 0 | 0 | 0 | 0 | 0 | 0 |
| KPHS_20120 | *phoQ* |  | 0 | 0 | 0 | 0 | 0 | 0 | 0 | 0 | 0 | 0 | 0 |
| KPHS_16340 | *pmrA* | Decrease in memberance permeatiblity | 0 | 0 | 0 | 0 | 0 | 1 | 0 | 0 | 0 | 0 | 1 |
| KPHS_16330 | *pmrB* |  | 2 | 2 | 2 | 2 | 1 | 1 | 1 | 1 | 1 | 1 | 16 |
|  | **Total** |  | 46 | 37 | 43 | 43 | 33 | 43 | 45 | 43 | 43 | 52 |  |
